# Effect of health intervention integration within women's self-help groups on collectivization and healthy practices around reproductive, maternal, neonatal and child health in rural India

**DOI:** 10.1371/journal.pone.0202562

**Published:** 2018-08-23

**Authors:** Niranjan Saggurti, Yamini Atmavilas, Akash Porwal, Janine Schooley, Rajshree Das, Narender Kande, Laili Irani, Katherine Hay

**Affiliations:** 1 Population Council, New Delhi, India; 2 Bill & Melinda Gates Foundation, New Delhi, India; 3 Project Concern International, San Diego, United States of America; Tulane University School of Public Health and Tropical Medicine, UNITED STATES

## Abstract

**Background:**

This study evaluates an eight-session behavior change health intervention with women’s self-help groups (SHGs) aimed to promote healthy maternal and newborn practices among the more socially and economically marginalized groups.

**Methods:**

Using a pre-post quasi-experimental design, a total of 545 SHGs were divided into two groups: a control group, which received the usual microcredit intervention; and an intervention group, which received additional participatory training around maternal, neonatal, and child health issues. Women members of SHGs who had a live birth in the 12 months preceding the survey were surveyed on demographics, practices around maternal, neonatal and child health (MNCH), and collectivization. Outcome effects were assessed using difference-in-difference (DID) methods.

**Results:**

Women from the SHGs with health intervention, relative to controls over time (time 1 to time 2), were more likely to: use contraceptive methods (DID: 9 percentage points [pp], p<0.001), have institutional delivery (DID: 9pp, p<0.05), practice skin-to-skin care (DID: 17pp, p<0.05), delay bathing for 3 or more days (DID: 19pp, p<0.001), initiate timely breastfeeding (DID: 21pp, p<0.001), exclusively breastfeed the child (DID: 27pp, p<0.001), and provide age-appropriate immunization (DID: 9pp, p<0.001). Additionally, women from the SHGs with health intervention when compared to the control group over time were more likely to report: collective efficacy (DID: 17pp, p<0.001), support through accompanying SHG members for antenatal care (DID: 8pp, p<0.05), receive a visit from SHG member within 2 days post-delivery (DID: 32pp, p<0.001), and receive reproductive, maternal, neonatal and child health information from an SHG member (DID: 45pp, p<0.001).

**Conclusion:**

Findings demonstrate that structured participatory communication on MNCH with women’s groups improve positive health practices. In addition, SHGs can reach a substantial proportion of women while providing an avenue for pregnant women and young mothers to be assisted by others in learning and practicing healthy behaviors, thus building social cohesion on health.

## Introduction

In India, the child mortality rate decreased by more than 62% between 1990 and 2015 (from 126 to 49 deaths per 1000 births) [1]. However, disparities exist both between and within states in the country, with northern states having the highest burden of child mortality, particularly among Uttar Pradesh, Haryana and Bihar [2,3]. Almost 41% of child deaths happen during the neonatal period, before the newborn completes his/her 28 days of life [2]. Most neonatal deaths in the northern region happen at home, and many can be avoided with changes in antenatal, delivery, and newborn care practices [4].

The spectrum of maternal, newborn and child health practices is varied and diverse including those that are one-time (e.g. immediate breastfeeding) and continuous (e.g. exclusive breastfeeding for at least 6 months)[5]; those that are supply dependent (e.g. consumption of iron and folic acid [IFA] tablets procured through public delivery) and those that depend more on learning and practice by mothers (e.g. skin-to-skin care, and delayed bathing) [6]. Essential maternal and newborn care practices coupled with timely care-seeking, especially during the first week of life, have been key to reducing maternal and neonatal deaths [7,8]. Many programs to-date that have focused around improving the delivery of health services have shown some success across geographies be it in improving quality of care at health facilities [9,10], increasing coverage with outreach through frontline workers [11,12] and strengthening the primary health care for maternal and child health [13,14]. Besides service delivery related barriers, the literature has shown that several harmful practices due to the prevailing social and cultural practices and gender norms continued to contribute to maternal and neonatal mortality in India [15,16]. Community based approaches have been an important and effective avenue to address those social and cultural practices while ensuring that health interventions reach the marginalized and poor in a community [17,18].

Demand or community-driven approaches also have a rich and growing evidence base where health interventions implemented with and through community groups–collectives, community based organisations (CBOs), self-help groups, mothers’ groups, among others–have resulted in improvements in health outcomes [7,19,20,21,22]. Self-help groups (SHG) are a form of women’s collectives aimed at empowering women and communities and addressing poverty. They draw on women’s social capital to promote shared goals[19,23]. SHGs aim at reaching the marginalized and economically disadvantaged, being scalable at low cost, and producing potentially wide-ranging and sustainable effects[24]. Intervening with SHGs have several advantages including reaching more socially marginalized women due to caste or other social inequalities [25], improving childcare and contraceptive use [26,27], reducing infant mortality and improving universal access to maternal health care services utilization [28,29]. A review by Lee et al found that community mobilization with high levels of community engagement (women’s groups) had a moderate effect in increasing health facility births and reducing perinatal mortality [30]. However, Bahl et al [31] demonstrated that the greatest impact on neonatal mortality and utilization of health facilities resulted from a combination of home visits by community health workers and mobilization with women’s groups. Further, the impact of a community mobilization programme through participatory women’s groups among the indigenous communities of Jharkhand and Odisha showed that neonatal mortality rate was 32% lower in intervention clusters over 3 years, and 45% lower in years 2 and 3 [32].

This growing body of evidence suggests that community mobilisation based health and development interventions, can improve outcomes and make them more sustainable, and achieve broader goals of addressing poverty and fostering well-being [14]. However, most evidence available to date is based on evaluation of small programs in select geographies. Literature examining the effects of health-integrated community mobilization programs at scale—especially, implemented with and through government programs are scarce. This paper addresses this gap in the literature and examines the effects of a large health intervention within SHGs on increasing the empowerment of women at the collective level while improving practices around maternal, neonatal and child health.

## Methods

### Study setting

Bihar is one of the most populous, poorest and lowest-performing states in terms of development outcomes in the nation, with great need for improvements in health services and outcomes. More than 50% of adult women are illiterate [33]; neonatal and infant mortality, maternal mortality, and fertility are all above the national average. Additionally, public health care access in Bihar is inadequate [34,35,36]; and there are challenges of access as well as quality of service delivery particularly for reproductive, maternal, newborn and child health, and nutrition.

#### Ananya’s demand-generation intervention

In 2011, the Bill and Melinda Gates Foundation implemented a set of innovations under the *Ananya* program in 8 districts of Bihar in order to reduce the maternal and neonatal mortality through a partnership with the Government of Bihar [37]. This initiative’s design combined both supply and demand-side interventions to improve reproductive, maternal, newborn and child health (RMNCH) services and outcomes. There was a focus on interventions delivered through frontline workers (FLWs), i.e., those health workers engaging in community outreach. Given that health interventions with community groups had demonstrated success in Jharkhand, Orissa, and Nepal [32,38,39], *Ananya* included an innovation with SHGs. The innovation–termed as Parivartan—entailed forming and nurturing 19,000 health-focused SHGs with women of reproductive age coming from the most marginalized communities, i.e., scheduled castes, scheduled tribes and *pasmanda* Muslims (considered to be a socially backward Muslim community). Health “integration” or layering included eight weekly cycles of participatory behavior communication using different thematic modules (see Table 1), on maternal, neonatal, child health and promoting collectivization processes facilitated by community health facilitators or *sahelis*. The aim was for women to learn about lifesaving maternal and newborn health practices in group meetings, and address lack of information and peer support to practice healthy behaviors.

**Table 1:** Overview of health integration intervention.

Session	Intervention content focus	Anticipated outcomes	Mode of delivery
**1**	Introduction Module	• Interrelation between Health and Livelihoods	• Banner with key messages and story of two women who had to invest loan amount on health emergency at the household level. • Consent letter by the Self- Help Groups to continue the discussion on Health, Nutrition and Sanitation
**2**	ANC and birth preparedness	• Early registration for ANC • Receipt of IFA tablets • Consumption of IFA tablets • Delivery in an institution	• Message Card and a story of a Musahar pregnant lady
**3**	Post natal care Focus of this module is on early breastfeeding and neonatal behaviors (delayed bathing, skin-to-skin care, clean cord care)	• Early initiation of breastfeeding • Not apply anything on the cord • Delay bathing for at least 72 hours • Practice skin-to-skin care	• A story a lady named Sarita who has just delivered
**4**	Exclusive breastfeeding and supplementary nutrition	• Exclusive breastfeeding for at least 6 months • Children above age 6 years given cereal based semi solid food	• Message Card and Picture Puzzle card
**5**	Routine immunization	• Children receive appropriate doses of intervention and in time • Children complete the receipt of DPT3 vaccine	• Banner with key messages and song (sohar–it’s a style of singing in Bihar which is sung on the occasion of birth of a child)
**6**	Family Planning	• Women use postpartum contraception Women continue to use contraception to prevent unwanted pregnancies	• Story Card with pictures
**7**	Personal Hygiene and safe storage if water at the household level	• Water borne diseases • Safe storage practices at household level • Hand washing during critical time	• Picture Cards and Song
**8**	Usage of Toilet and Garbage Management	• Use of toilet • Safe disposal of child’s stool	• Faeces mapping (participatory mapping process where in the areas where the villagers go for open defecation were marked using yellow colour; after pouring water, villagers were able to visualise which all places could be contaminated due to open defecation). Picture Card and Song

**Note: *Example of a story from Session 4*:** Meena went to deliver her baby at the local hospital with her husband and mother-in-law. Shortly thereafter, she delivered a healthy girl. The nurse put the baby on Meena’s chest immediately and the baby had her first breastmilk. The baby was named Khushboo. At three months of age, Khushboo’s aunt came to visit her. As it was the middle of summer, the aunt encouraged the mother to begin feeding the baby water as breastmilk exclusively would not be sufficient. Meena explained to the aunt that Khushboo doesn’t need any supplement besides her own milk for the first four months.

#### Control condition

Groups in the control condition were part of government nurtured SHGs, which provide financial literacy and savings support and services, combined with unstructured health and social messages. In the control groups, the program was delivered by a range of functionaries with responsibilities for different functions including community mobilizers and community resource persons who were equivalent to Sahelis in the Parivartan intervention. The control condition did not have any structured focus on maternal, neonatal and child health practices.

### Study design and sampling

A two-armed quasi-experimental design was used to evaluate the impact of the structured health intervention with SHGs on maternal, neonatal and child health practices. A total of 1182 groups were sampled from rural areas of 35 blocks in eight districts of Bihar, India from April to June 2013, and the same groups were followed over a period of 12 months for this evaluation.

A two-stage cluster sampling design was used to select the participating groups from all eight districts. At the first stage, 35 of 67 blocks (the initial project area) were randomly selected with the use of random numbers generated using MS-Excel. Of the 35 selected blocks, in 27 blocks, Parivartan program was implemented. In the remaining eight blocks, the government worked with SHGs on small savings and credit, and those groups were selected as controls. In the second stage, the SHGs were systematically selected within each selected block. The SHG lists that were available with the program were used as a sampling frame for the selection of SHGs. The first SHG was selected using a random number followed by every n^th^ SHG chosen from the list. By the 12th-month of follow-up, the Parivartan program in one of the districts was discontinued, resulting in 7 districts with 24 blocks (Table 2).

**Table 2:** Matched sample size of self-help groups between Time 1 and Time 2.

Sample Sizes	Time 1 - Complete sample	Time 1 - Matched sample (to time 2)^#^	Time 2
Number of districts*	8	7	7
Number of blocks	35	24	24
Number of groups (panel)		545	545
Number of women belonging to common groups (analytical sample)		1,539 (S+H: 1,095; S only: 444)	937 (S+H: 720; S only: 217)

* one district was excluded from the study at time 2 due to non-cooperation from the groups

^**#**^ Intervention in one of the districts is discontinued due to lack of enough groups and cooperation from the local program agency

The number of women required for the study was estimated to detect the difference between the intervention and control arms in at least three key family health and sanitation indicators (i.e., at least three antenatal care visits, institutional delivery and use of toilets) with 95% confidence and 5% of margin of error. Further, the number of SHGs selected were estimated based on the number of eligible women needed for the evaluation and the estimated number of eligible women available per SHG. In total, 1182 groups (810 Parivartan program and 372 control groups) were sampled for the study. The ratio of groups selected for intervention and control groups were proportional to the size of the groups formed by Parivartan program and the government of Bihar, respectively.

### Study population

Group leaders belonging to this quasi-experimental design (n = 568 intervention groups, n = 176 control groups) were surveyed at times 1 and 2; and all eligible women from these groups were surveyed simultaneously. Eligible women from the groups included women belonging to scheduled castes/ scheduled tribes or pasmanda muslims, aged above 18 years and had given a live birth in the 12 months preceding the survey. Both the group leaders and the women participants had to provide consent to participate in this study. Of the 1182 groups who were approached to study in time 1, 979 groups could be identified, and all the group leaders from these groups agreed to participate in the study. The high participation rate of group leaders was attributable to the fact that the groups were nurtured through programs (either through Parivartan or the government). Of the 979 groups, 744 had at least one female member who was eligible to be part of the study.

#### Study retention

Of the 744 groups participating in the time 1 assessment, only 73% were able to complete the study at 12-month period. Reasons for loss to follow-up (199 groups) was due to the non-functionality of a group at time 2, and/or scale up with formation of other governmental groups.

### Data collection

Trained female research teams approached identified groups for recruitment at both time points. Group leaders indicating interest and willingness to participate provided written informed consent and were interviewed using a semi-structured questionnaire that assessed the group’s functionality and the members’ characteristics. Group leaders were then asked about any members who had a live birth in 12 months preceding the survey. Once eligibility was ascertained, that member of the group was approached privately for participation in the survey, and women who provided consent participated in the survey. All literate women who agreed to participate in the survey had signed the consent forms. In case of illiterate women, the information in the consent form was read out to them by the investigator. Those who agreed to participate gave their thumb impression to indicate their consent. All the eligible women who had given consent were interviewed separately using a structured survey questionnaire. A copy of the consent was provided to respondents for their records. Surveys were administered in a face-to-face interview format with research staff asking questions and noting the responses of the women who participated in the survey.

As part of ensuring data quality in the survey, each member of the data collection team was supervised and monitored by a supervisor whose primary role was to observe the smooth functioning of the data collection and upload the data to a centralized server on daily basis. Once the data were uploaded, they were analysed to check for patterns, errors in key indicators daily by the key research team member in the office. Any discrepancies/outliers noted were cross-checked with the field team the next day to understand the data better. The daily monitoring and feedback to the research team, ensured the generation of good quality data, kept the investigators under check and only allowed the highest quality data collectors to remain onboard while minimizing the field research teams’ access to the raw data beyond the field work days thereby reducing the potential for data tampering.

Following the baseline assessment with the group leaders and eligible women from the groups, the groups from the intervention areas received structured health messaging through a trained peer worker (known as ‘Saheli’). Follow-up survey in the same groups were conducted at 12-month follow-up. Eligible women were again identified from those groups for interview. There was no woman who was interviewed twice in this study, as one of the eligibility criteria included the live birth within past 12 months. Data were collected by research investigators using mini-laptops. A user-written computer program in CSPro (v4.0) (developed for the US Census Bureau, Washington D.C., USA) was used to present both English and Hindi questions simultaneously so as to reduce the error in data collection, time in data entry, and to allow for real time data uploads for rapid management of data for analyses [40]. No monetary incentive was provided to participants, but they were given information on incentives provided by health programs and the available financial services from the government. All study procedures and consent forms were reviewed and approved by the Institutional Review Board of the Population Council.

### Variables

The sociodemographic characteristics (e.g., age, parity, occupation, literacy, caste and duration of group association) were assessed via single item measures.

#### Non-health outcome variables

Collectivization was the primary non-health outcome variable measuring the empowerment of women at the collective level. It was assessed via the measures that describe efficacy, agency, action and cohesion around maternal and child health; these were made up of multiple indicators comprising a composite index. Collective efficacy is defined as the belief of the self-help group in its power to work together to bring positive changes around health. It was measured on the basis of four questions: How confident are you that the members of your community can work together to achieve the following goals? (a) Speaking up against the existing norms imposed by elderly or other groups on issues around mother and child health; (b) Demanding services from healthcare facilities when they refuse support; (c) Claiming rights/schemes from the government; (d) Increase the safe practices around mother and child health (for eg., ensuring the delivery at hospital, immunization of children, seeking antenatal care). Responses to these questions included: not at all confident (coded 1), somewhat confident (coded 2), very confident (coded 3), and completely confident (coded 4). Using responses to these questions, a scale ranging from 1 to 4 was created by taking the mean of the responses. Low collective efficacy was defined as falling between 1 and 2.499 on this scale; whereas high collective efficacy was defined as falling between 2.5 and 4.

Collective agency includes a cluster of questions related to SHG members assisting other members to seek/demand healthcare services or services from local administrative agencies. It was measured based on three questions: In the past six months, have you negotiated with or stood up against the following to help a fellow community member? (a) Health care center staff e.g. doctor, nurse; (b) Frontline workers e.g. accredited social health activists, Anganwadi workers, auxiliary nurse midwife; (c) Local administration (e.g. police, civil supplies etc.). Responses to these questions included yes (coded 1) and no (coded 0). Using responses to these questions, a scale ranging from 0 to 1 was created by taking the mean of the responses. Low collective agency was defined as falling between 0 and 0.499 on this scale; whereas high collective agency was defined as falling between 0.5 and 1.

Collective action refers to the strategic and organized activities of SHGs to increase the members’ presence or enact its agenda for change. It was measured on the basis of eight questions around whether or not the group came together to demand the following: (1) better health services for mother and child from local health center; (2) services/schemes meant for the poor e.g. for the Janani Suraksha Yojana (JSY) scheme; (3) on-time delivery of incentives from the government; (4) opening of bank accounts: (5) availability of family planning services; (6) supply of safe drinking water; (7) supply of sufficient water for household purposes; and, (8) supply of sanitation services e.g. latrine facilities. Responses to these questions included yes (coded 1) and no (coded 0). Using responses to these questions, a scale ranging from 0 to 1 was created by taking the mean of the responses. Low collective action was defined as falling between 0 and 0.499 on this scale; whereas high collective action was defined as falling between 0.5 and 1.

Group Cohesion for health was measured on the basis of three direct questions: (a) whether any SHG member accompanied the respondent for antenatal care (yes/no); (b) whether any SHG member made a visit within 2 days after delivery (yes/no); (c) whether the respondent received RMNCH information from any SHG member other than through the intervention sessions (yes/no).

#### Health outcome variables

The key health outcome variables were assessed via a single item questions for each of the following indicators: (1) use of modern methods of contraception (mothers with 6–11 months old children); (2) use of modern spacing methods (mothers with 6–11 months old children); (3) four or more antenatal care visits during pregnancy; (4) consumption of IFA tablets/syrup for 100 or more days; (5) place of delivery; (6) immediate (within 2 days) post-natal visit by a health worker; (6) skin-to-skin care to keep newborn baby warm; (7) delayed bathing for 3 or more days; and, (8) timely initiation of breastfeeding. Exclusive breastfeeding was calculated using two questions: whether the child was breastfed in 24 hours prior to the survey (yes/no), and, was the child given any liquid and food items in 24 hours prior to the survey (yes/no)? Age-appropriate immunization was calculated using information on different types of vaccinations that the child is likely to receive at different ages. For example, questions were asked that whether the child received vaccination on the dosages of polio, BCG, DPT, measles and vitamin A.

#### Independent variables

The primary independent variable was intervention group, SHGs with health intervention or control. The receipt of health intervention was classified as receiving: if the SHG was included in the health intervention group or not. All the SHGs selected in the study group had completed the 8-module session before the time 2 survey. SHGs in the control group did not receive any structured health intervention.

### Statistical analysis

Unadjusted analyses were conducted using summary statistics. Bivariate analyses in the form of t-tests and chi-squares were conducted to assess differences on demographics and program input variables at time 1 and time 2: (1) by intervention group. Any characteristics identified as significantly different between groups were considered as potential covariates in respective adjusted models. Regression-adjusted difference-in-difference model in which an individual’s outcome was regressed against a dummy variable, indicating whether a woman is part of the SHG with health integration, and a series of health and non-health outcome variables were presented in results. All statistical analyses were done using STATA version 13.0.

## Results

The average number of months since the SHGs formed was 8±2 months with significant variation by intervention and control groups (Table 3). Little more than 10% of the groups in both intervention and control arms were exclusive scheduled caste or tribal groups. Women in the health intervention groups received more visits from the Saheli (as she was responsible for 7 groups) as compared to women in the control groups (where each Saheli was responsible for 23 groups). Mean age of participants in the baseline was 25±5 years. Only 10% of the women had formal education, and about two-fifths of the women reported being engaged in economic activity. There were differences between the groups in the age, education and economic activity engagement at both times 1 and 2. No significant differences in characteristics of women (age, education, occupation, number of children ever born) or groups (number of literate members per group, number of months since the groups were formed) were seen between those self-help groups who dropped out and who remained in follow-up surveys; nor were any differences observed between women in self-help groups who did and did not participate in intervention during the assessment period.

**Table 3:** Background characteristics of the groups & Socio-demographic characteristics of the women.

	Total (n = 545)	Intervention groups (n = 374)	Control groups (n = 171)	p-value
Group characteristics (Time 1)				
Age of the groups (mean +/- SD)^$^ (in months)	8.0(±1.8)	7.9(±1.9)	9.0(±1.3)	<0.001
Group membership size (mean +/- SD)	14.9(±2.1)	14.9(±2.2)	14.7(±2.1)	0.972
Number of literate members per group (mean +/- SD)	1.3(±2.4)	1.3(±2.4)	1.2(±2.5)	0.083
Exclusive SC/ST groups (%)		12.4	10.6	
Health intervention	**NA**	High intensity	Low intensity	**NA**
Saheli (health guide) support ratio	**NA**	7 groups	23 groups	**NA**
**Individual characteristics (Time 1)**	**N = 1539**	**N = 1095**	**N = 444**	
Average age of the women (Mean +/- SD) (in years)	25.3(±4.9)	25.1(±4.8)	26.0(±5.0)	0.002
Literacy (%)	11.5	13	8.3	0.012
Engaged in economic activity (%)	46.7	42.7	56.5	<0.001
Average number of children ever born (Mean +/- SD)	3.0(±1.7)	2.9(±1.7)	3.3(±1.7)	0.845
**Individual characteristics (Time 2)**	**N = 935**	**N = 718**	**N = 217**	
Average age of the women (Mean +/- SD) (in years)	26.2(±4.6)	26.0(±4.7)	26.6(±4.4)	0.971
Literacy (%)	14.2	15.9	9.0	0.008
Engaged in economic activity (%)	40.8	37.5	51.2	<0.001
Average number of children ever born (Mean +/- SD)	3.2(±1.6)	3.0(±1.6)	3.7(±1.6)	<0.001

p value denotes the probability value of Chi square test

$ is the standard deviation

Regression adjusted difference-in-difference estimates for reproductive and newborn health outcomes showed consistent, statistically significant increases across time for SHGs with health integration intervention (p<0.001) (Table 4). The net increase with health integration among SHGs for timely initiation of breastfeeding was 20 percentage points (p<0.05), and a similar result noted in case of delayed bathing (p<0.05). Exclusive breastfeeding showed statistically significant increase over time for SHGs with health integration than without health integration (DID: 26 percentage points, 95% CI: 9–44, p<0.05). Similarly, age appropriate immunization among children under one year showed a consistent increase over time (DID: 9 percentage points, 95% CI: 1–20, p<0.05). The net increase in use of contraception among mothers with 6–11 months child is 9 percentage points (95% CI: 1.3, 17.2, p<0.001). The change over time were similar for SHGs with and without health integration on maternal health indicators such as 4 or more antenatal care visits, consumption of IFA tablets and postnatal visit from a health worker, indicating no net increases due to health integration intervention.

**Table 4:** Estimated effect of health intervention on reproductive, maternal, neonatal and child health outcomes.

	Intervention groups			Control groups			Adjusted^#^ DID (95% CI)
	Time 1	Time 2	p-value	Time 1	Time 2	p-value	
**Maternal health outcomes**							
Proportion of individuals who went for 4+ antenatal care visits	10.2	14.2	0.012	5.4	9.7	0.047	-0.4 (-6.2, 5.5)
Proportion of individuals reporting consumption of IFA tablets/syrup for 100+ days	5.9	12.8	<0.001	6.3	8.2	0.415	4.9 (-1.1, 10.8)
Proportion of individuals who went for institutional delivery	60.2	70.7	<0.001	61	63.6	0.525	8.8 (-0.1, 17.8)*
Percentage women who visited by a health worker within 2 days after delivery	32.4	47.5	<0.001	27.7	47.9	<0.001	-4.6 (-13.6, 4.4)
**Newborn care** ^^^							
Skin-to-skin care (keeping newborn warm)	36.6	62.3	<0.001	31.8	42.3	0.162	17.0 (-0.5, 34.1)*
Delayed bathing for 3+ days	18.7	50.4	<0.001	14.7	26.9	0.053	19.2 (3.8, 34.6)**
Timely initiation of breastfeeding	65.0	82.9	<0.001	84.1	80.7	0.574	20.5 (5.7, 35.3)***
**Child care**							
Exclusive breastfeeding ^^^	32.9	50.4	<0.001	46.6	37.2	0.222	26.7 (9.4, 44.1)***
Fed solid/semi-solid food ^**@**^	76.4	73.4	0.219	73.5	65	0.053	4.7 (-5.3, 14.6)
Age appropriate immunization	42.9	55.3	<0.001	52.5	56.2	0.365	9.1 (1.0, 19.6)**
**Reproductive health outcomes**							
Use of modern methods of contraception^**@**^	11.9	23.6	<0.001	13.2	15.8	0.456	9.3 (1.3, 17.2)**
Use of modern spacing methods	2.3	9.3	<0.001	0.6	4.1	0.002	3.3 (-0.4, 7.0)*
Use of traditional methods for spacing	1.4	6.3	<0.001	0.9	0.9	0.979	4.9 (1.9, 7.9)***

# Difference in difference is adjusted for background characteristic of respondents across intervention and control groups: age, parity, occupation, literacy, caste, duration of group association

p values at ***, **, * defines significance level at 1%, 5% and 10% respectively

^ Calculated for mothers with child aged 0–5 months (Time 1 = 334; Time 2 = 330)

@ Calculated for mothers with child aged 6–11 months (Time 1 = 1200; Time 2 = 605)

Regression adjusted (for age, education and occupation) difference-in-difference estimates for collective efficacy showed statistically significant improvement in collective efficacy (DID: 17 percentage points; 95% CI: 8–26; p<0.001), group cohesion around SHG member accompanying the target woman to health clinics for antenatal care (DID: 8 percentage points; 95% CI: 1–14; p<0.001), and making post-natal visits within first two days (DID: 32 percentage points; 95% CI: 24–39; p<0.001) among groups that had intervention than the control groups (Table 5). The proportion of women receiving RMNCH information from SHG members over time has increased significantly in intervention groups than those in control groups (DID: 45%; 95% CI: 38–53%; p<0.001).

**Table 5:** Estimated effect of health intervention on collectivization and cohesion to achieve health goals.

Community mobilization indicators	Intervention groups			Control groups			Adjusted^#^ DID (95% CI)
	Time 1	Time 2	p-value	Time 1	Time 2	p-value	
**Collectivization outcomes**							
Collective efficacy (High)	38.8	56.1	<0.001	51.8	51.0	0.833	16.9 (7.5, 26.2)***
Collective agency (High)	13.2	8.5	<0.001	14.1	15.6	<0.001	-5.4 (-11.6, 0.6)
Collective action (High)	28.5	33.4	0.025	36.0	42.9	0.090	-0.7 (-9.5, 8.1)
**Cohesion outcomes**							
SHG member accompanied for ANC	5.3	29.5	<0.001	17.6	33.6	<0.001	7.7 (1.0, 14.4)**
SHG member made visit within 2 days after delivery	6.5	52.8	<0.001	34.5	48.4	0.001	31.8 (24.1, 39.3)***
Received RMNCH information from SHG member	12.6	68.2	<0.001	30.2	40.1	0.011	45.4 (37.6, 53.2)***

#Difference in difference is adjusted for background characteristic of respondents across intervention and control groups: age, parity, occupation, literacy, caste, duration of group association

p values at ***, **, * defines significance level at 1%, 5% and 10% respectively

## Discussion

The findings indicate that behavior change communication on life-saving maternal and newborn care practices with women’s groups worked, i.e. it led to a substantial improvement in maternal, newborn and child healthcare practices among most marginalized women in India. The results are consistent with earlier findings on the effects of interventions that address maternal and newborn health through women’s collectives [23,41]. These findings highlight the importance of imparting a systematic approach using participatory behavior change communication with women’s groups as a method to promote maternal, neonatal and child behaviors in order to further reduce maternal and child mortality.

Results of this study further demonstrate the effects of peer support, networking, and cohesion in achieving increases in health practices among women [23,42]. Prior research from other countries and in India indicates that health intervention with groups reduces neonatal mortality and improves universal access to services [38,39,43,44]; the current study extends these findings to indicate increased peer support among members works for MNCH practices.

Notably, there wasn’t much improvement in collective agency and action to achieve the health goals. Much of the literature that provides an understanding around agency and action within health is available in HIV prevention programs [45,46,47,48]. Studies on HIV education programs with groups provide evidence that agency and action are the advanced stages of collectivization that can be achieved over time, and through intentional focus on building agency from the beginning of the program [17,46,49]. Similar to HIV prevention programs, the health integration intervention in self-help groups of women and their mobilization seems to be a process. The first step in the process involves an increased sense of being together, coined collective efficacy. Next, individual SHG members begin advocating for their, their fellow members’ and the community’s needs with administrative authorities. Notably, this process is tedious and requires concerted efforts on the part of the group members. The plausible reason for decline in agency within intervention areas (though the difference-in-difference is not significant) may be because the self-help groups formed in the state are relatively young, women are young mothers, and the intervention focus is on the individual members around the practice of positive health behaviors of their own rather than addressing the needs of a larger community. And this presents a need for long-term research to study the progression from efficacy to agency and action of women in SHGs and its interplay with health and empowerment interventions (economic and social) in influencing the desired and sustainable outcomes.

Findings suggest that newborn care practices—that are broadly one-time point and supply-independent practices changed significantly. Maternal health indicators like the number of antenatal care visits, consumption of IFA tablets, and the postnatal visit from a health worker did not change significantly. The lack of impact of the intervention in these specific areas of maternal health highlights the need for improved agency and action, wherein members of the group (or) the SHG leadership makes the health systems accountable to make services and supplies more accessible. For example, the consumption of IFA tablets/syrup for 100 or more days require that those many tablets were supplied to women during pregnancy. Post-hoc analyses of the current study data suggested that, consumption of IFA tablets/syrup for 100 or more days is greater among women who were members of SHGs with health intervention than their counterparts. However, the proportion receiving 100+ IFA tablets/syrup is very low across the study geographies.

Although findings offer important insights around the effect of health intervention within SHGs, the results must be interpreted in the light of certain study limitations. Firstly, the groups in the comparison arm are little older than the groups in the intervention arm. Additionally, the groups in the intervention arm were formed as SHGs promoting savings and health behavior change while groups in the comparison arm were formed as savings and credit groups without any plans to build health training. However, the groups in the intervention arm established similar elements as the government SHGs within a year, reducing the variability and potential for bias when comparing the two types of groups. Secondly, the input, process and outcome indicators were based on self-reports, which are vulnerable to social desirability and recall biases. The potential for bias was kept to a minimum by collating and comparing information from other sources for matching of results. The recall bias was reduced by recruiting women who have given birth to a child one year prior to the survey. The potential for social desirability bias was reduced by recruiting different women in each round of the survey, although the groups are longitudinal. Thirdly, the concepts around collectivization used in the survey were borrowed from the studies conducted among key population groups within HIV program [17], and therefore, may not exactly be relevant to the context and lives of most marginalized women in the general population. However, the reliability scores calculated for collectivization indicators suggests that they are applicable even within the context of SHGs.

## Conclusion

Participatory behavior communication on MNCH with women’s groups appears to be an effective community-based approach. This intervention, delivered by a trained community worker, offers a potentially sustainable approach to reaching most-marginalized population groups in rural India and possibly elsewhere. The opportunity to involve SHGs in low-income settings for this effort cannot be overstated. This is certainly a meaningful strategy in India, given the volume of SHGs in rural India and the different kinds of community groups that are being implemented under the national rural livelihood mission [50]. The intervention may further benefit with linking available government services to the SHG platform wherein, women become change agents within the environment and bring accountability to various services at the local level. The intervention is also promising as a sustainable approach as it utilizes an existing group-based platform to build on women’s social capital and peer networks to build women’s access to information, services, and agency for health.

## Supporting information

S1 File This is the S1 File questionnaire. This is the bilingual (Hindi and English) study questionnaire.
(PDF)
