# Supplementary material for: Effect of health intervention integration within women's self-help groups on collectivization and healthy practices around reproductive, maternal, neonatal and child health in rural India
Source: PLoS One. 2018 Aug 23;13(8):e0202562. doi: 10.1371/journal.pone.0202562 (PMC6107172; doi:10.1371/journal.pone.0202562)
Supplement: S1 File — This is the bilingual (Hindi and English) study questionnaire. (PDF) [file pone.0202562.s001.pdf]

.

CONFIDENTIAL
ONLY FOR RESEARCH PURPOSES

PROJECT PARIVARTAN: परिवर्तन परियोजना

WOMEN SURVEY QUESTIONNAIRE: महिला प्रश्नावली

ध्यान दें: सिर्फ उन महिलाओं का साक्षात्कार करना है जो:

1. समूह की सदस्य हैं
2. अनुसचित जाति, अनुसूचित जनजाति, या पिछड़े मुस्लिम समुदाय से हैं
3. जिनके सबसे छोट बच्चे की उम एक साल या उससे कम है

IDENTIFICATION OF VILLAGE गांव की पहचान	
**DIST.** DISTRICT NAME: जिला	DISTRICT CODE: जिले का कोड
**BLOCK.** BLOCK NAME: ब्लॉक	BLOCK CODE: ब्लॉक का कोड
**VILLAGE.** VILLAGE NAME: गांव	VILLAGE CODE: गांव का कोड
**GID. GROUP ID** सामुदायिक समूह का **नाम और** पहचान कोड	
**LN.** LINE NUMBER OF THE WOMAN IN THE LIST OF GROUP समूह की सूची में महिला की लाइन नंबर	
**TYPE.** TYPE OF THE GROUP **समूह का प्रकार**	
Parivartan परिवर्तन..............................1<br>Other अन्य………………………………..3	Jeevika जीविका..2
**INT_CODE** INTERVIEWER NAME साक्षात्कारकर्ता का नाम:	INTERVIEWER ID साक्षात्कारकर्ता का आईडी:
**VDAY: दिन**	**VMONTH: महीना**
**VYEAR: साल**	

BLOCK 1:SOCIO-DEMOGRAPHIC DETAILS

#	Question	Answers	Codes	Skip to
**101**	How old are you now?<br>आपकी उम्र कितनी है	Age in completed years<br>उम्र पूर्ण वर्षों में		
**102**	What is your husbands' age?<br>आपके पति की उम्र कितनी है	Age in completed years<br>उम्र पूर्ण वर्षों में		
**103**	What was your age at the time of marriage?<br>शादी के समय आपकी उम्र क्या थी	Age in completed years<br>उम्र पूर्ण वर्षों में		
**104**	What was your age at your first child birth?<br>आपके पहले बच्चे के जन्म के समय आपकी उम्र क्या थी	Age in completed years<br>उम्र पूर्ण वर्षों में		
**105**	Can you read and write?<br>क्या आप पढ़-लिख सकती हैं	Cannot read and write पढ़ लिख नहीं सकती<br>Can read only सिर्फ पढ़ सकती है<br>Can sign only केवल दस्तखत कर सकती है<br>Can read and write पढ़ व लिख सकती है	1<br>2<br>3<br>4	► 107<br>यदि प्रश्न संख्या 105

				में 1 कोड हुआ है तो प्रश्न संख्या 107 पर जाएँ
**106**	What is the highest standard you have completed?<br>आपने कहां तक पढ़ाई की है	Standard<br>कक्षा		
**107**	Can your husband read and write?<br>क्या आपके पति पढ़-लिख सकते हैं	Cannot read and write पढ़ लिख नहीं सकते<br>Can read only सिर्फ पढ़ सकते है<br>Can sign only केवल दस्तखत कर सकते है<br>Can read and write पढ़ व लिख सकते है	1<br>2<br>3<br>4	► 109<br>यदि प्रश्न संख्या 107 में 1 कोड हुआ है तो प्रश्न संख्या 109 पर जाएँ
**108**	What is the highest standard your husband has completed?<br>आपके पति ने कहां तक पढ़ाई की है	Standard<br>कक्षा		
**109**	What is your (women's) main occupation?<br>आपका (महिला का) मुख्य व्यवसाय क्या है	Wage laborer दैनिक मजदूर<br>Skilled/ semi-skilled worker कुशल /अर्ध कुषल कामगार<br>Service (Govt. / Private) नौकरी (सरकारी/निजी)<br>Self-employed/ Business स्वरोजगार /व्यापार<br>Agriculture/cultivator कृषि/कृषक<br>Unemployed-has intention to work, but not working currently बेरोजगार-कार्य करना चाहती है, पर अभी कोई कार्य नहीं कर रही है<br>Housewife-has no intention to work गृहिणी- कार्य करने का कोई इरादा नहीं है	1<br>2<br>3<br>4<br>5<br>6<br>7	
**110**	What is your husbands' occupation<br>आपके पति का व्यवसाय क्या है	Wage laborer दैनिक मजदूर<br>Skilled/ semi-skilled worker कुशल/अर्ध कुषल कामगार<br>Service (Govt. / Private) नौकरी (सरकारी/निजी)<br>Self-employed/ Business स्वरोजगार/व्यापार<br>Agriculture/cultivator कृषि/कृषक<br>Unemployed बेरोजगार	1<br>2<br>3<br>4<br>5<br>6	
**110a**	Where does your husband work ?<br>आपके पति कहाँ काम करतें हैं?	Within our village हमारे गाँव में<br>In the town, but within district शहर में पर जिले के अन्दर<br>Outside the district but within state जिले के बाहर लेकिन बिहार प्रदेश के अन्दर<br>Outside the state बिहार प्रदेश के बाहर<br>In Mumbai/Thane मुंबई/ठाणे में	1<br>2<br>3<br>4<br>5	If <3, skip to 110c<br>यदि उत्तरदाता प्रश्न संख्या 110a

				में 3 से कम कोड करें तो प्रश्न संख्या 110c पर जाएँ
**110b**	How often does he visit here ?<br>आपके पति कितने अंतराल पर घर आतें हैं?	Once in three months तीन महीने में एक बार<br>Once in six months छे महीने में एक बार<br>Once a year साल में एक बार<br>More than a year एक साल से ज्यादा में<br>No regular schedule (but visits every year) कोई निश्चित समय नहीं;(पर हर साल आते हैं)<br>No regular schedule (hasn't visited in the past year) कोई निश्चित समय नहीं (पिछले साल में एक बार भी नही आये)	1<br>2<br>3<br>4<br>5<br>6	
**110c**	What is your household's monthly income (including your husband's and your own earnings) ?<br><br>आपके घर की (आपके पति और आपकी) मासिक आमदनी कितनी है?	Write income<br>कमाई लिखें		
**111**	Do you belong to a scheduled caste, a scheduled tribe, or other backward caste?<br>क्या आप अनुसुचित जाति, अनुसचित जनजाति या अन्य पिछडे वर्ग से है	Scheduled caste अनुसुचित जाति<br>Scheduled tribe अनुसचित जनजाति<br>OBC पिछडा वर्ग<br>Others अन्य<br>Pasmunda Muslims पास्मंदा मुसलमान	1<br>2<br>3<br>4<br>5	
**112**	What is your religion?<br>आपका धर्म क्या है	Hindu हिन्दु<br>Muslim मुस्लिम<br>Sikh सिख<br>Christian इसाई<br>Buddhist बुद्धिस्ट<br>Jain जैन<br>Others अन्य	1<br>2<br>3<br>4<br>5<br>6<br>7	
**113**	How many children do you have?<br>आपके कितने बच्चे हैं<br>(सभी जीवित बच्चों की संख्या दर्ज करें।	**A.** Total number of sons बेटों की कुल संख्या<br><br>**B.** Total number of daughters बेटीयों की कुल संख्या		

	उन बच्चों को भी शामिल करें जो उत्तरदाता के साथ नहीं रह रहे हैं।)	**C.** Total number of children कुल बच्चों की संख्या		
**113_**<br>1	Have you ever given birth to a boy or girl who was born alive but later died ? (Died within one year)<br><br>क्या आपने कभी लड़की अथवा लड़के को जन्म दिया हैं जो जीवित पैदा हुआ पर बाद में मर गया? (सिर्फ ऐसे बच्चे जिनकी उम्र मृत्यु के समय एक साल से कम थी उनके बारें में बताएं<br>)<br><br>If NO, PROBE : Any baby who cried or showed signs of life but did not survive<br><br>गहराई से पूछें: ऐसा बच्चा जो रोया अथवा जीवित होने के आसार थे पर बचा नहीं	Yes हां<br>No नहीं	1<br>2	►<br>113_<br>4<br>यदि प्रश्न संख्या 113_ 1में 2 कोड हुआ है तो प्रश्न संख्या 113_ 4 पर जाएँ
**113_**<br>2	How many such births have you given?<br><br>आपने ऐसे कितने बच्चे को जन्म दिया?	__ (Write down number) संख्या लिखिए		
**113_**<br>3	What was the age of the last child when he/she died ?<br>सबसे बाद में जिस बच्चे की मृत्यु हुई एउसकी मृत्यु के वक़्त उम्र क्या थीा	days (if age is in months, convert to days and write)<br>(दिन में संख्या लिखिए अथवा बच्चे की उम्र अगर महीनों में है तो दिन में बदल कर लिखें)		
**113_**<br>4	Have you ever had a pregnancy that miscarried, was aborted or ended in a stillbirth ?<br>क्या आपको कभी ऐसी गर्भावस्था हुई है जिसमे बच्चा गिर गया, गिरा दिया गया अथवा मरा हुआ पैदा हुआ?	Yes हां<br>No नहीं	1<br>2	►11<br>4<br>यदि प्रश्न संख्या 113_ 4 में 2 कोड हुआ है तो प्रश्न संख्या

				114 पर जाएँ
**113_5**	How many such pregnancies have you had?<br>आपको इस तरीके के कितने गर्भ हुए हैं?	Write down number __<br>संख्या लिखिए		
**114**	Are you currently pregnant?<br>क्या आप अभी गर्भवती हैं	Yes हां<br>No नहीं	1<br>2	► 201<br>यदि प्रश्न संख्या 114 में 2 कोड हुआ है तो प्रश्न संख्या 201 पर जाएँ
**115**	How many months pregnant are you now?<br>आप अभी कितने माह से गर्भवती हैं	Months महीने	_______	
116	Are you registered for this pregnancy with ANM, ASHA or AWW ?<br><br>क्या आप इस गर्भावस्था के लिए एएनएमए आशा और आंगनवाडी कार्यकर्ता के साथ पंजीकृत है?	Yes…हाँ 1<br>No…नहीं .2		

Now I will ask you some questions about the pregnancy related to your youngest child as well as some questions about other aspects related to your youngest child. अब मैं आपसे आपकी सबसे छोटी आयु की संतान के बारे में कुछ प्रश्न पूछूंगी

BLOCK 2: ANTENATAL CARE प्रसवपूर्व देखभाल से सम्बंधित प्रश्न

अब मैं आपसे आपकी पिछली गर्भावस्था के दौरान हुई देखभाल के बारे में कुछ प्रश्न पूछन चाहूंगी

#	Question	Answers	Codes	Skip to
**201**	Did you see any health provider (ASHA,ANM, AWW or some other heathcare provider) for antenatal care for this pregnancy?<br>क्या आपने **पिछली** गर्भावस्था के लिए प्रसव पूर्व देखभाल के लिए किसी भी स्वास्थ्य प्रदाता **से** मुलाकात की?	Yes हां<br>No नहीं	1<br>2	► 205<br>यदि प्रश्न संख्या 201में 2 कोड हुआ है तो प्रश्न संख्या 205 पर जाएँ

**202**	Where did you receive antenatal care for the	**HOME** घर		

	last pregnancy?<br>आपने **पिछली** गर्भावस्था के लिए प्रसव पूर्व देखभाल कहां से प्राप्त **कीा थी?**	Your home आपका घर<br>Parents' home आपके माता पिता का घर<br>Other home अन्य घर<br>**PUBLIC MEDICAL SECTOR**<br>**सरकारी स्वास्थ्य सेक्टर**<br>Govt/municipal hospital<br>सरकारी / म्युनिसिपल अस्पताल<br>Govt dispensary सरकारी डिस्पेंसरी<br>PHC प्राईमरी स्वास्थ्य केन्द्र<br>Sub-center उप केन्द्र<br>Anganwadi centre आंगनवाडी केन्द्र<br>**NGO / PVT. MEDICAL HOSPITAL**<br>**गैर सरकारी संस्था / निजी अस्पताल**	A<br>B<br>C<br><br><br><br>D<br>E<br>F<br>G<br>H<br><br>I	
202a	Whom did you see?<br>**प्रसवपूर्व देखभाल के लिए** आपने किसको दिखाया था / मुलाकात की?<br>**MARK ALL THAT APPLY**<br>**जो लागू हो सभी को कोड करें**	PUBLIC DOCTOR सरकारी डाकअर A<br>PRIVATE DOCTOR प्राइवेट डाक्टर ………………..….B<br>ANM ए एन एम C<br>NURSE नर्स ……………………………………………………….…D<br>LHV स्थानीय स्वास्थ्य कार्यकर्ता ……………………………………E<br>OTHER HEALTH PERSONNEL अन्य स्वास्थ्य कार्यकर्ता F<br>DAI/Trained dai दाई / प्रशिक्षित दाई G<br>ASHA आशा H<br>AWW आंगनवाडी कार्यकर्ता ………………………... I<br>OTHER (SPECIFY) अन्य स्पष्ट करें X		
**203**	How many months pregnant were you when you first received antenatal care (advise/treatment) for this pregnancy?<br>**पिछली गर्भावस्था के लिए जब आपको पहली बार प्रसव पूर्व देखभाल मिली तब आप कितने महीने के गर्भ से थी?**	Months महीने	______	
**204**	How many times did you go for antenatal check up during this pregnancy?<br>आपको **पिछली** गर्भवस्था के दौरान कितनी बार प्रसव पूर्व देखभाल मिली थी?	Number of times<br>प्रसवपूर्व देखभाल की संख्या	______	
**205**	Did any woman from your community group accompany you during (any of) your antenatal visits?<br>क्या आपके सामुदायिक समूह की कोई महिला किसी भी प्रसव पूर्व देखभाल के लिए आपके साथ गयी थी	Yes हां<br>No नहीं<br>There was no group at that time<br>उस समय कोई समूह नहीं था<br>Respondent was not member of the group at that time	1<br>2<br>3<br><br>4	 ► 208<br><br>► 208<br>► 208

		उस समय उत्तरदाता समूह की सदस्य नहीं थी		यदि प्रश्न संख्या 205 में 1 से ऊपर कोड हुआ है तो प्रश्न संख्या 208 पर जाएँ

			Yes	No	206b
**206**	During your last pregnancy, did anyone from your community group tell you about the following signs of pregnancy complications? पिछले गर्भावस्था के दौरान क्या किसी ने आपको गर्भावस्था की निम्नलिखित परेषानियों के बारे में बताया था? **प्रत्येक के बारे में अलग अलग पूछें**	A. Swelling of hands, feet and Face हाथ पैर और चेहरे पर सूजन आना	1	2	
		B. Paleness/giddiness/weakness पीलापन/चक्कर/कमजोरी	1	2	
		C. Visual disturbances धुंधला दिखना	1	2	
		D. Excessive fatigue अत्यधिक थकान	1	2	
		E. Weak or no movement of fœtus बच्चे का धीरे घूमना या चक्कर लगाना	1	2	
		F. Abnormal position of fœtus बच्चे की असामान्य स्थिति	1	2	
		G. Excessive vomiting अत्यधिक उलटी होना	1	2	
		H. Hypertension/ High BP उच्च ब्लड प्रेषर	1	2	
		I. Jaundice पीलिया	1	2	
**206b**	Who told you about the complications ? (Multiple response question) आपको इन परेशानियों के बारें में किसने बताया<br>A. Saheli सहेली<br>B. Community leaders समूह के मुखिया<br>C. Community group member समूह के साथी महिला ने<br>D. ASHA आशा<br>E. ANM ए एन एम्<br>F. AWW अन्गंवादी वर्कर<br>X. Other अन्य	J. Excessive bleeding अत्यधिक रक्तस्राव	1	2	
		K. Convulsions कपकपी आना/दौरा पड़ना (बुखार वाला नहीं)	1	2	
		L. Prolonged Labor प्रसव के दौरान ज्यादा देर तक दर्द होना	1	2	
		M. Obstructed labour प्रसव के दौरान रूक रूक कर दर्द होना	1	2	

	Mark All That Apply □□<br>से ज्यादा उत्तर संभव है				

**207**	Did anyone from your community group tell you where to go if you had any pregnancy complications?<br>क्या आपके समूह मे से किसी ने आपको यह बताया था कि गर्भावस्था से संबंधित कोई परेषानी होने पर कहॉ जाना हैं	Yes हां<br>No नहीं<br>There was no group at that time<br>उस समय कोई समूह नहीं था<br>Respondent was not member of the group at that time<br>उस समय उत्तरदाता समूह की सदस्य नहीं थी	1<br>2<br>3<br>4	
**208**	Did you experience any of the pregnancy complications during your last pregnancy?<br>क्या आपने पिछली गर्भावस्था के दौरान किसी भी प्रकार की परेषानी का अनुभव किया	Yes हां<br>No नहीं	1<br>2	► 212<br>यदि प्रश्न संख्या 208 में 2 कोड हुआ है तो प्रश्न संख्या 212 पर जाएँ

			209		210	
**209**<br><br>**210**	What complications did you experience?<br>आपने कौन सी परेशानियों का अनुभव किया<br><br>And did you receive treatment for the problem ?<br>क्या आपने इस दिक्कत का उपचार करवाया ?<br><br>**प्रत्येक के बारे में अलग अलग पूछें**		**Did you have this problem**<br>**क्या आपको यह परेशानी हुई?**		**Did you seek treatment ?**<br>क्या आपने इस दिक्कत का उपचार करवाया ?	
			**Yes हाँ**	**No नहीं**	**Yes हाँ**	**No नहीं**
		AA. Swelling of hands, feet and Face<br>हाथ पैर और चेहरे पर सूजन आना	1	2	1	2
		B. Paleness/giddiness/weakness<br>पीलापन/चक्कर/कमजोरी	1	2	1	2
		C. Visual disturbances<br>धुंधला दिखना	1	2	1	2
		D. Excessive fatigue<br>अत्यधिक थकान	1	2	1	2
		E. Weak or no movement of fœtus<br>बच्चे का धीरे घूमना या चक्कर लगाना	1	2	1	2
		F. Abnormal position of fœtus<br>बच्चे की असामान्य स्थिति	1	2	1	2
		G. Excessive vomiting	1	2	1	2

		अत्यधिक उलटी होना				
		H. Hypertension/ High BP<br>उच्च ब्लड प्रेषर	1	2	1	2
		I. Jaundic पीलिया	1	2	1	2
		J. Excessive bleeding<br>अत्यधिक रक्तस्राव	1	2	1	2
		K. Convulsions<br>कपकपी आना/दौरा पड़ना (बुखार वाला नहीं)	1	2	1	2
**211**	Where did you seek treatment (if participant stated receiving treatment for any of the problems)?<br>आपने इलाज कहां करवाया (यदि इलाज कराया गया तो)	Government Hospital सरकारी अस्पताल<br>Private hospital निजी अस्पताल<br>Rural Medical Practioner झोलाछाप चिकित्सक<br>Other (specify) अन्य (स्पष्ट करें)	A<br>B<br>C<br>X			

**212**	During delivery, did you experience any of the following problems?<br>प्रसव के दौरान क्या आपने इनमे से किसी भी परेषानी का अनुभव किया था?<br>**प्रत्येक के बारे में अलग अलग पूछें**		**Yes हाँ**	**No नहीं**
		A. Premature labour समय से पहले दर्द	1	2
		B. Excessive bleeding अत्यधिक रक्तस्राव	1	2
		C. Prolonged labour ज्यादा समय तक दर्द	1	2
		D. Obstructed labour रूक रूक कर दर्द	1	2
		E. Breech presentation बच्चे की असामान्य स्थिति	1	2
		F. Convulsion/High B.P शारीरिक ऐठन/उच्च ब्लडप्रेषर	1	2

Check Box: प्रश्न संख्या 213 तभी पूछें यदि 209A से 209I तक किसी एक में भी "हॉ" जवाब आया हो या 212A से 212F तक किसी एक में "हॉ" जवाब आया हो। यदि इन सभी प्रश्नों में "नहीं" जवाब आ या हो तो प्रश्न संख्या 216 पर जाऍं

**213**	Did you discuss the complications during pregnancy/ delivery with any of your community group members?<br>क्या आपने गर्भावस्था या प्रसव के दौरान हुई किसी भी परेषानी के बारे में समूह के सदस्यों से चर्चा **की थी**?	Yes हां<br>No नहीं<br>There was no group at that time<br>उस समय कोई समूह नहीं था<br>Respondent was not member of the group at that time<br>उस समय उत्तरदाता समूह की सदस्य नहीं थी	1<br>2<br>3<br>4	 ► 216<br>► 216<br>► 216<br>यदि प्रश्न संख्या 213 में 1 से ऊपर कोड हुआ है तो प्रश्न संख्या 216 पर जाएँ

**214**	Did you receive any support from community group members on the problems that occured during pregnancy/ dleivery?<br>क्या आपको गर्भावस्था **या प्रसव के दौरान हुई परेशानी में** समूह के सदस्यों से किसी भी प्रकार की मदद मिली?	Yes हां<br>No नहीं	1<br>2	► 216<br>यदि प्रश्न संख्या 214 में 2 कोड हुआ है तो प्रश्न संख्या 216 पर जाएँ
**215**	What type of support did you receive?<br>आपको समूह के सदस्यों से किस प्रकार का सहयोग मिला<br><br>[Multiple responses possible]<br>**एक से ज्यादा जवाब संभव है**	Information about the problem<br>समस्या के बारे में सूचना मिली<br>Suggestion to see a qualified health professional<br>कुशल चिकित्सक को दिखाने की सलाह मिली<br>Help in going to the health center, and treatment<br>समूह के सदस्यों ने इलाज के लिए स्वास्थ्य केन्द्र तक जाने, और इलाज लेने में मदद किया (जैसे कि इलाज के लिए पैसे दिया, स्वास्थ्य केंद्र तक जाने में वाहन का इंतजाम किया, स्वास्थ्य केंद्र तक साथ में गए, इत्यादि)<br>Community members negotiated and convinced the family members for treatment<br>समूह के सदस्यों ने परिवार वालों को इलाज करवाने के लिए सहमत किया<br><br>Community group members informed the ASHA/ANM and ensured referral<br>**समूह के सदस्यों ने आशा अथवा ए एन एम् को सूचित किया जिससे वह डॉक्टर अथवा अस्पताल भेज दें**<br><br>Other अन्य (स्पष्ट करें)	A<br><br>B<br><br>C<br><br>D<br><br>E<br><br>X	
**216**	During this pregnancy, were you given an injection to prevent you and the baby from getting tetanus?<br>गर्भावस्था के द्वौरान आपको और आपके बच्चे को टेटनेस से बचाने के लिए क्या कोई इंजेक्षन दिया गया था ?	Yes हां<br>No नहीं	1<br>2	► 218<br>यदि प्रश्न संख्या 216 में 2 कोड हुआ है तो प्रश्न संख्या 218 पर जाएँ
**217**	During the pregnancy, how many times did you get a tetanus injection?<br>गर्भावस्था के द्वौरान कितनी बार टेटनेस	Number of times<br>टेटनेस के इंजेक्षन की संख्या	______<br>___	

	का इंजेक्षन लिया था?			
**218**	Did you receive any Iron Folic Acid (IFA) tablets during this pregnancy? (Show tablets)<br>गर्भावस्था के दौरान आपको आयरन फोलिक एसिड (ताकत की लाल गोली) टेबलेट मिली थी ? **(गोली दिखाएँ)**	Yes हां<br>No नहीं	1<br>2	► 220 यदि प्रश्न संख्या 218 में 2 कोड हुआ है तो प्रश्न संख्या 220 पर जाएँ
**218**a	How many tablets did you receive in total during the whole of your last pregnancy?<br>आपकी इस गर्भावस्था के दौरान आपको आयरन फोलिक एसिड (ताकत की लाल गोली) की कुल कितनी टेबलेट मिली थी ?	____ (Write number received)		
**219**	During the whole pregnancy, for how many days did you take the tablets?<br>अपनी इस पूरी गर्भावस्था के दौरान टेबलेट का सेवन आपने कितने दिनों तक किया?	Number of days<br>**दिन**		
**219a**	**<u>Other than the iron tablets that I showed you</u>**, did you take iron syrup, iron capsules or any other iron formulation from a chemist or doctor or anywhere else?<br>क्या आपने कोई अन्य आयरन सिरप/आयरन कैप्सूल या कोई अन्य आयरन की चीज़ किसी मेडिकल डॉक्टर/मेडिकल दुकान से खरीदीधमिली?	Yes हां<br>No नहीं	1<br>2	
**220**	During the last pregnancy, did you get supplementary food from the Anganwadi centre?<br>पिछली गर्भावस्था के दौरान जब आपने चाहा तब आपको आगनवाडी केंद्र से राशन(पोषाहार) मिला था?	Yes हां<br>No नहीं	1<br>2	

**220A**	In the last 3 months of the last pregnancy, how many times did an FLW come and meet you in your house ?<br><br>प्रसव के अंतिम 3 महीनो के दौरान थ्ॅ कितनी बार आपके घर पर आपसे मिलीघ	Number of times<br>Don’t know/don’t remember…. 98<br><br>संख्या<br>पता नहीं/ याद नहीं 98		

**221**	During the last pregnancy, did you receive advice on the following at least once either from the health workers (ASHA, ANM, AWW, Doctor) or other women from your community?<br>पिछली गर्भावस्था के दौरान क्या आपने निम्नलिखित पर कम से कम एक बार स्वास्थ्य कार्यकर्ताओं (आषा, एएनएम, आंगनवाडी कार्यकर्ता, डाक्टर) या अपने समुदाय की अन्य महिलाओं द्वारा सलाह प्राप्त **की?**<br><br>**Who provided the information?].**<br>आपको यह सलाह किससे मिली?<br><br>Health worker (ASHA/ ANM/ AWW/ Doctor) स्वास्थ्य कार्यकर्ता (आषा, एएनएम, ए डब्ल्यु डब्ल्यु, डाक्टर) ....1<br><br>Community group member<br>समुदायिक समूह के सदस्य............................2<br><br>Both		Yes<br>हाँ	No<br>नहीं	**222**<br>Who gave you this advice? (1 – HW, 2 – Community group member, 3 – Both)<br>□□ सलाह आपको किसने दी (1 - हेल्थ कार्यकर्ता ; 2 समूह के सदस्य; 3 दोनों नेघ
		A. How to recognize danger signs of pregnancy गर्भावस्था के खतरे की पहचान कैसे करेंगे	1	2	1 2 3
		B. How to recognize the danger signs at the time of delivery (for eg., prolonged labor, excessive bleeding, etc.) प्रसव के समय खतरे के संकेत की पहचान कैसे करेंगे (उदाहरणः लंबी प्रसव पीडा, अत्यधिक रक्तस्राव)	1	2	1 2 3
		C. Keeping the new born baby warm नवजात षिषु को गर्म रखना	1	2	1 2 3
		D. Placing the infant unclothed on the mother's chest with skin to skin contact under a blanket or some clothing नवजात षिषु को बिना कोई कपड़ा पहनाए, मां की छाती से सटा कर किसी कंबल या ओढ़ने के अंदर रखना।	1	2	1 2 3
		E. Keep soft clean cloth for drying the baby and a separate clean cloth for wrapping the newborn baby साफ सुथरा कपड़ा नवजात षिषु को पोछने के लिए और अलग से साफ कपड़ा नवजात षिषु को लपेटने के लिए	1	2	1 2 3 9
		F. Have a disposable delivery kit (DDK) एक डिलिवरी किट	1	2	1 2 3 9
		G. Use a clean blade to cut the cord नाल काटने के लिए एक साफ ब्लेड का प्रयोग	1	2	1 2 3 9
		H. Breastfeeding the child immediately /within half an hour नवजात षिषु को तुरंत/आधे घंटे के अंदर स्तनपान कराना	1	2	1 2 3- 9

	दोनो.......................3<br><br>Other अन्य<br>...............9	I. Information about recommended vaccinations to the child नवजात षिषु के लिए बताए गये टीकाकरण के बारे में जानकारी	1	2	1 2 3 9
		J. Family planning for delaying your next child अगले बच्चे में देरी के लिए परिवार नियोजन	1	2	1 2 3 9
		K. Timings when you need to wash hands कब–कब हाथ धोने की जरूरत होती है	1	2	1 2 3 9
**223**	Did you attend any Village Health Nutrition Days (VHNDs) during your pregnancy?<br><br>क्या आपने अपनी गर्भवस्था के दौरान गाँव के स्वास्थ्य पोषण दिवस में<br>भाग लिया था॰	Yes हां<br>No नहीं	1<br>2		►301<br>यदि प्रश्न संख्या 223 में 2 कोड हुआ है तो प्रश्न संख्या 301 पर जाएँ
**224**	When was the last time that you attended VHND meeting?<br><br>आपने अंतिम बार गाँव के स्वास्थ्य पोषण दिवस में कब भाग लिया था?	Number of months ago _____________<br>कितने महीने पहले			

BLOCK 3: BIRTH PREPAREDNESS

Now, i would like to ask you about **preparations or decisions** you made for the delivery of [CHILD NAME].
अब मैं आपसे कुछ प्रश्न आपकी प्रसव के बारे में कि गयी तैयारियों और निर्णयों के बारे में पूछना चाहूंगी

#	Question	Answers	Codes	Skip to
**301**	Prior to delivery, did you plan or intend to deliver [CHILD NAME] at home or in a healthcare facility?<br>बच्चे को जन्म देने से पहले, क्या आपने यह योजना बनाई थी या सोचा था कि जन्म घर मे या स्वास्थ्य सुविघा केंद्र मे हो?	At home घर पर<br>In a healthcare facility स्वास्थ्य सुविघा केंद्र में<br><br>Did not plan नही सोचा था	1<br>2<br><br>3	 <br><br>►401<br>यदि प्रश्न संख्या 301 में 3 कोड हुआ है तो प्रश्न संख्या 401 पर

				जाएँ
**302**	Did you discuss plans for your delivery with anyone in your family? If yes, with whom?<br>क्या आपने अपने प्रसव की योजना के बारे में अपने परिवार वालों से साथ बात की थी ? यदि हॉ, तो किससे?<br>**(CHECK ALL THAT RECORDED)**	Yes, with husband हॉ, पति से<br>Yes, Mother-in-law हॉ, सास से<br>No नहीं	1<br>2<br>3	
**303**	Did you discuss plans for your delivery with anyone in the community group?<br>क्या आपने अपने प्रसव/डिलीवरी की योजना के बारे में आपने सामुदायिक समूह वालों से साथ बात की थी?	Yes हां<br>No नहीं	1<br>2	►305 यदि प्रश्न संख्या 303 में 2 कोड हुआ है तो प्रश्न संख्या 305 पर जाएँ
**304**	If yes, with whom did you discuss?<br>यदि हॉ, तो किससे आपने चर्चा कि?	With Saheli only केवल सहेली के साथ<br>With most members of our group हमारे समूह के अधिकांश सदस्यों के साथ<br>Both saheli and group members<br>सहेली और समूह सदस्यों, दोनों के साथ	1<br>2<br>3	
**Check Box: if 301 = 2 then skip to 306 अगर 301 में "2" कोड हुआ हो तो 306 पर जाएँ**				

<u>अब मैं आपसे कुछ प्रश्न घर पर प्रसव करने के लिए की गयी तैयारियों के बारे में पूछूंगी</u>

305	Did you or anyone in your family make any preparations for delivery at home to…?<br>आप या आपके परिवार मे से किसी ने **घर पर प्रसव के लिए** कुछ तैयारिया कि थी. . . . ?	**Yesहॉ**	**Noनहीं**
	**A.** Identify and arrange for a trained birth attendant, such as an ANM, DAI to be present during childbirth?<br>**प्रसव** के लिए एक कुशल सहायक जैसे कि एएनएम या दाई की पहचान की और व्यवस्था की जो बच्चे के जन्म के दौरान उपस्थिति रहें ?	1	2
	**B.** Obtain a clean delivery kit (Disposable Delivery Kit (DDK))?<br>साफ डिलीवरी किट प्राप्त करना (डिस्पोजेबल डिलीवरी किट)<br>**IF YES, SKIP TO "E" अगर "हाँ" तो "E" पर जाएँ**	1	2
	**C.** Obtain a new blade to cut the cord?<br>नाल काटने के लिये नया ब्लेड लेना	1	2
	**D.** Obtain a new/clean thread to tie the cord?<br>नाल को बांधने के लिये नया/साफ धागा लेना	1	2
	**E.** Obtain clean cloth for drying the baby?<br>बच्चे को सुखाने के लिये साफ कपडा लेना	1	2

	**X.** Other? (SPECIFY) अन्य (उल्लेख करें)	1	2

AFTER ASKING 305X SKIP TO 401
इस प्रश्न को पूछने के बाद 401 पर जाएँ

Now I would like to ask you about anything specific that you did to **prepare for a delivery at a facility**
अब मैं आपसे कुछ प्रश्न स्वास्थ्य केंद्र पर प्रसव कराने के लिए की गयी तैयारियों के बारे में पूछूंगी

306	Did you or anyone in your family make any preparation for delivery in a health facility to…?<br>क्या आप या आपके परिवार में किसी ने भी कोई तैयारी कि.....<br>**DO NOT READ LIST. CODE ALL MENTIONS IN FIRST COLUMN.**<br>**सूची को न पढ़े. जो जवाब महिला दे, उसे पहले कॉलुम में नोट करें**<br>**THEN READ EACH ITEM BELOW (A-E) THAT WASN'T MENTIONED AND CODE RESPONSE IN SECOND COLUMN.**<br>**जब महिला अपनी तरफ से सारे जवाब दे चुकी हो तो वह जवाब जो महिला ने ना दिया हो, उसे पूछें**	**Yes**<br>हाँ	**No**<br>नहीं
	**A.** Save money for the delivery डिलीवरी के लिये पैसा बचा कर रखा था	1	2
	**B.** Identify health facility for the delivery? डिलीवरी के लिये स्वास्थ्य सुविधा की पहचान की थी?	1	2
	**C.** Identify transportation to go to health facility? स्वास्थ्य सुविधा तक जाने के लिये यातायात की पहचान की थी?	1	2
	**D.** Identify a person to accompany you to the healthcare facility? स्वास्थ्य सुविधा तक आपके साथ जाने के लिये किसी व्यक्ति की पहचान की थी ?	1	2
	**X.** Other? (SPECIFY) अन्य (उल्लेख करें)	1	2

BLOCK 4: DELIVERY AND POST NATAL CARE

अब मैं आपसे कुछ प्रश्न प्रसव और बच्चे के जन्म के समय आपके अनुभवों के बारे में पूछूंगी

**#**	**Question**	**Answers**	**Codes**	**Skip to**
**401**	Where did you deliver [CHILD NAME]?<br>आपने बच्चे को कहाँ जन्म दिया था?<br><br>**CODE ONE ONLY**<br>सिर्फ एक ही उत्तर संभव है<br><br>**IF UNABLE TO DETERMINE IF A HOSPITAL, HEALTH CENTRE, OR CLINIC IS PUBLIC OR PRIVATE MEDICAL SECTOR, WRITE THE NAME OF THE PLACE.**<br><br>यदि यह नहीं पता कर पाते की	**HOME घर**<br>Your home आपका घर<br>Parents' home आपके माता पिता का घर<br>Other home अन्य घर<br>**PUBLIC MEDICAL SECTOR**<br>**सरकारी स्वास्थ्य सेक्टर**<br>Govt/municipal hosp. सरकारी/म्युनिसिपल अस्पताल<br>Govt dispensary सरकारी डिस्पेंसरी<br>UHC/UHP/UFWC शहरी स्वास्थ्य केन्द्र/षहरी स्वास्थ्य योजना/षहरी परिवार कल्याण केन्द्र<br>CHC/Rural hospital कम्यूनिटी हेल्थ सेन्टर/ग्रामीण अस्पताल<br>PHC प्राईमरी स्वास्थ्य केन्द्र<br>Sub-center उप केन्द्र<br>Anganwadi/ICDS centre आंगनवाडी/आय सी डी एस केन्द्र<br>**NGO SECTOR गैर सरकारी सेक्टर**<br>NGO/Trust/Clinic गैर सरकारी संस्था/ट्रस्ट/क्लीनिक<br>**PVT. MEDICAL SECTOR निजी मेडिकल सेक्टर**<br>Pvt.hospital /maternity home निजी अस्पताल	 1<br>2<br>3<br><br><br>4<br>5<br>6<br><br>7<br>8<br>9<br>10<br><br>11<br><br>12	

	अस्पताल, हेल्थ सेण्टर अथवा क्लिनिक (सरकारी या निजी) गए थे तो जगह का नाम लिखें ____________	Other Pvt. sector facility अन्य निजी सेक्टर स्वास्थ्य सुविधायें	13	
**402**	Who assisted with the delivery of [CHILD'S NAME]?<br>(बच्चे का नाम) की डिलीवरी किसने करायी थी ?<br><br>CIRCLE ALLTHAT APPLY<br>एक से ज्यादा उत्तर संभव हैं	Qualified doctor कुषल डाक्टर<br>Staff nurse/LHV स्टाफ नर्स/एलएचवी<br>ANM ए एन एम<br>Untrained Dai अकुशल दाई<br>ASHA/ AWW आषा/आंगनवाडी कार्यकर्ता<br>Members of community समुदाय के सदस्य<br>Unqualified doctor/RMP अशिक्षित डाक्टर/आर एम पी<br>No one कोई नहीं<br>Other अन्य<br>Trained DAI कुशल दाई	A<br>B<br>C<br>D<br>E<br>F<br>G<br>H<br>X<br>I	
**403**	Who were all along with you at the time of delivery?<br>प्रसव के समय आपके साथ कौन से लोग थे?<br>एक से ज्यादा उत्तर संभव हैं	Husband/relatives पति/रिष्तेदार<br>Friends/neighbours मित्र/पड़ोसी<br>Front line workers फ्रंट लाईन कार्यकर्ता<br>Community group members सामूदायिक समूह के सदस्य<br>No one कोई नहीं	A<br>B<br>C<br>D<br>E	
404	Was the delivery normal or caesarean or assisted?<br>क्या प्रसव सामान्य रूप से हुआ या ऑपरेशन से हुआ या फिर औजार की सहायता से हुआ था?	Normal/ सामान्य<br>Caesarean/ ऑपरेषन<br>Assisted/forceps or vacuum pump/ सहायता जैसे फोरसेप्स अथवा वच्चुम पंप के साथ	1<br>2<br>3	
**Check Q401: प्रश्नसंख्या 401 की देखें। प्रश्नसंख्या 405 से लेकर 408 तक सिर्फ उन्ही महिलायों से पूछें जिनकाप्रसव किसी स्वास्थय केंद्र (सरकारी या निजी) में हुआ था। अगर प्रसव घर पर हुआ था तो 409 पर जाएँ**				
**405**	Were you promised money or an incentive for delivery of your baby in a healthcare facility through the JSY program?<br>क्या आपको जननी सुरक्षा योजना के अर्न्तगत स्वास्थ्य केन्द्र में डिलीवरी कराने पर पैसा या पुरस्कार का वादा किया गया था ?	Yes हां<br>No नहीं	1<br>2	
**406**	Did you get the amount?<br>क्या आपको पैसा मिला ?	Yes हां<br>No नहीं	1<br>2	►409 यदि प्रश्न संख्या 406 में 2 कोड हुआ है तो प्रश्न

				संख्या 409 पर जाएँ
**407**	How much was the payment received?<br>आपको कितने पैसे मिले थे	INR रूपये	______	
**407a**	How was the payment made? Was it by cash or cheque or directly transferred to your bank or other account?<br>आपको पैसे कैसे मिले थे? क्या यह नकद में हुआ/चैक/सीधा आपके बैंक में या अन्य किसी के बैंक में हुआ ?	Cash नकद .... 1<br>Cheque चेक .... 2<br>Directly transferred to account<br>सीधा आपके खाते में....................................... 3<br>Other (specify) ....<br>अन्य (स्पष्ट करें)...		
**408**	How long after the delivery did you receive the money?<br>डिलीवरी के कितने दिनों के बाद आपको पैसे मिले थे?	Number of days<br>दिनों की संख्या	______	
**409**	After delivery, did anyone (health worker / comunity member) place [CHILD NAME] unclothed in skin-to-skin contact to your chest or abdomen?<br>प्रसव के बाद क्या किसी स्वास्थ्य कार्यकर्ता या समूह के सदस्यों ने (बच्चे का नाम) को बिना कोई कपडा पहनाए आपकी छाती या पेट पर रखा था?	Yes हां<br>No नहीं	1<br>2	
**Check Q401: प्रश्न्संख्या 401को देखें| प्रश्न्संख्या 409a से लेकर 409c तक सिर्फ उन्ही महिलायों से पूछें जिनका प्रसव घर में हुआ था|**				
**409a**	Since you delivered at home, did you make any arrangments for money and transportation in case there was an emergency?<br>क्योंकि आपने घर मे ही प्रसव करवाया, क्या आपने आपातकालीन स्थिती के लिए पैसे अथवा वाहन का इंतज़ाम किया था?	Yes हाँ No नहीं ►410	यदि प्रश्न संख्या 409a में 2 कोड हुआ है तो प्रश्न संख्या 410 पर जाएँ	
**409b**	Made arrangements for money in case of emergency<br><br>आपातकालीन स्थिती के लिए पैसे का इंतज़ाम किया	1 हाँ 2 नहीं		

	था?			
**409c**	Made arrangemnets for transportation in case of emergency<br><br>आपातकालीन स्थिथि के लिए वाहन का इंतज़ाम किया?	1 हाँ 2 नहीं		
**410**	Who cut the unbilical cord?<br>नाभि की नाल किसने काटी थी?	Qualified doctor कुषल डाक्टर<br>Staff nurse/LHV स्टाफ नर्स/एलएचवी<br>ANM ए एन एम<br>Trained Dai कुशल दाई<br>Untrained dai अकुशल दाई<br>ASHA/ AWW आषा/आंगनवाडी कार्यकर्ता<br>Group members समूह के सदस्य<br>Unqualified doctor/RMP अशिक्षित डाक्टर/आर एम पी<br>No one कोई नहीं<br>Other अन्य	1<br>2<br>3<br>4<br>10<br>5<br>6<br>7<br>9<br>8	
**411**	What instrument was used to cut the umbilical cord?<br>नाभि की नाल काटने के लिये किस चीज का प्रयोग किया गया था ?<br><br>**PROBE:** Was it new or used and from where did you get it?<br>क्या वह नया था या उसको पहले भी इस्तेमाल किया गया था और आपने कहॉ से प्राप्त किया था ?	New blade from home घर से नया ब्लेड<br>New blade from DDK kit डिस्पोजेबल डिलीवरी किट से नया ब्लेड<br>Used blade from home घर से पुराना प्रयोग किया गया ब्लेड<br>Blade from doctor/ nurse/ ANM डाक्टर/नर्स/ एएनएम द्वारा दिया गया ब्लेड<br>Blade from TBA दाई द्वारा दिया गया ब्लेड<br>Scissor कैंची<br>Other (specify) अन्य (उल्लेख करें)<br>Don't know मालुम नहीं		1<br>2<br>3<br>4<br>5<br>6<br>8<br>9
**412**	What was used to tie the cord?<br>नाल को बांधने के लिये क्या प्रयोग किया गया था ?<br><br>**PROBE:** Was it new or used and from where did you get it?<br>क्या वह नया था या उसको पहले भी इस्तेमाल किया गया था और आपने कहॉ से प्राप्त किया था<br><br>**CODE ONE ONLY**<br>**सिर्फ एक उत्तर संभव है**	New thread from home घर से नया धागा<br>New thread from DDK kit डिस्पोजेबल डिलीवरी किट से नया धागा<br>Used thread from home घर से पुराना प्रयोग किया गया धागा<br>Thread from doctor/ nurse/ ANM डाक्टर/नर्स/एएनएम द्वारा दिया गया धागा<br>Thread from TBA दाई द्वारा दिया गया धागा<br>Cord clip कॉर्ड क्लिप<br>Other (specify) अन्य (उल्लेख करें)<br>Don't know मालुम नहीं	1<br>2<br>3<br>4<br>5<br>6<br>8<br>9	
**413**	Was anything applied to the cord after cutting and tying?<br>क्या काटने और बांधने के तुरन्त बाद नाल पर कुछ लगाया गया था ?	Yes हां<br>No नहीं	1<br>2	
**414**	Was anything applied to the umbilicus after the cord dropped off?<br>क्या जब नाल गिर गयी थी तो नाभी पर कुछ लगाया गया था ?	Yes हां<br>No नहीं	1<br>2	
**CHECK BOX: IF answers to both 413 and 414 are 2, then skip to 416.**				

अगर 413 और 414 दोनों में "नहीं" जवाब है तो 416 पर जायें				
**415**	What was applied to the cord after cutting and tying,?<br>आपने नाल को काटने और बांधने के बाद नाल पर क्या लगया था ?<br><br>**CODE ALL THAT APPLY**<br>**एक से ज्यादा उत्तर संभव हैं**	Powder/lotion/ointment (dettol/savoln, etc.)<br>पाउडर/लोषन/मलहम(डेटाल/सेवलान इत्यादि)<br>Alcohol/spirit एल्कोहल/स्पिरिट<br>Mustard oil सरसों का तेल<br>Sindoor सिन्दूर<br>Pure ghee षुद्ध धी<br>Boric powder बोरिक पावडर<br>Gentian violet (neeli dawai) निली दवाई<br>Talcum powder शरीर पर लगाया जाने वाला पावडर<br>Chlorohexidine क्लोरोहेक्सिडाइन<br>Cowdung गाय का गोबर<br>Others (specify) अन्य (उल्लेख करें)	A<br><br>B<br>C<br>D<br>E<br>F<br>G<br>H<br>I<br>J<br>X	
**415a**	What was applied to the umbilicus after the cord dropped off?<br>आपने नाल गिरने के बाद नाभि पर क्या लगया था ?	Powder/lotion/ointment (dettol/savoln, etc.)<br>पाउडर/लोषन/मलहम(डेटाल/सेवलान इत्यादि)<br>Alcohol/spirit एल्कोहल/स्पिरिट<br>Mustard oil सरसों का तेल<br>Sindoor सिन्दूर<br>Pure ghee षुद्ध धी<br>Boric powder बोरिक पावडर<br>Gentian violet (neeli dawai) निली दवाई<br>Talcum powder शरीर पर लगाया जाने वाला पावडर<br>Chlorohexidine क्लोरोहेक्सिडाइन<br>Cowdung गाय का गोबर<br>Others (specify) अन्य (उल्लेख करें)	A<br><br>B<br>C<br>D<br>E<br>F<br>G<br>H<br>I<br>J<br>X	
**416**	After delivery, was the baby wiped dry, wiped with a wet cloth, or bathed?<br>प्रसव के बाद क्या बच्चे के शरीर को पोंछ कर सूखा बनाया गया, गीले कपड़े से पोंछा गया या उसे नहलाया गया?	Wiped dry पोछ कर सुखाया गया..........................1<br>Wiped with a wet cloth……………………………2<br>गीले कपड़े से पोंछा गया<br>Bathed नहलाया गया था.....................................3<br>Don't know पता नहीं 98		

#	Question	Answers
**417**	How soon after the delivery was [CHILD NAME] given (his/her) first bath?<br>(बच्चे का नाम) को जन्म के कितने समय बाद पहली बार नहलाया गया था ?<br>**अगर एक घंटे से कम समय बताती है तो घंटे में "00" कोड करें और अगले प्रश्न पर जाएँ।**<br>**अगर 24 घंटे से कम समय बताती है तो जवाब को घंटे में कोड करें और अगले प्रश्न पर जाएँ**<br>**अगर 24 घंटे से ज्यादा समय बताती है तो घंटे में 99 भरकर, जवाब को दिन में दर्ज करें।**	Hours घण्टे<br>________________<br>Days दिन<br>________________

**418**	Did you ever breastfeed [CHILD NAME]?<br>क्या आपने (बच्चे का नाम) को कभी अपना दूध पिलाया ?	Yes हां<br>No नहीं	1<br>2	►421<br>यदि प्रश्न संख्या 418 में 2 कोड हुआ है तो प्रश्न संख्या

				421 पर जाएँ
**419**	How long after birth did you first put [CHILD NAME] to the breast?<br>आपने (बच्चे का नाम) को पहली बार अपना दूध कब पिलाया था ?<br>**अगर एक घंटे से कम समय बताती है "00" कोड करें और अगले प्रश्न पर जाएँ।**<br>**अगर 24 घंटे से लम समय बताती है तो जवाब को घंटे में कोड करें और अगले प्रश्न पर जाएँ**<br>**अगर 24 घंटे से ज्यादा समय बताती है तो घंटे में 99 भरकर, जवाब को दिन में दर्ज करें।**	Immediately/Within half an hour<br>**तुरंत/ आधे घंटे के अन्दर**<br>H. Hours घंटे ___________<br>D. Days दिन ____________	0	
420	Was [CHILD NAME] given anything other than breast milk within the first day?<br>क्या आपने (बच्चे का नाम) को पहले दिन में अपने दूध के अलावा कुछ और भी दिया था	Yes हां<br>No नहीं	1<br>2	 ►421A<br>**यदि प्रश्न संख्या 420 में 2 कोड हुआ है तो प्रश्न संख्या 421a पर जाएँ**

**421**	What was [CHILD NAME] given?<br>(बच्चे का नाम) को क्या दिया गया था ?<br><br>**PROBE:** Anything else?<br>कुछ और?<br>**एक से ज्यादा जवाब संभव हैं**	Milk Other Than Breast Milk मॉ के दूध के अलावा कोई और दूध (जानवर का दूध)<br>Plain Water सादा पानी<br>Sugar Or Glucose Water चीनी या ग्लूकोज का पानी<br>Gripe Water ग्राईप वाटर<br>Sugar-Salt-Water Solution चीनी नमक पानी का घोल<br>Fruit Juice फलों का रस<br>Infant Formula नवजात फार्मूला<br>Tea चाय<br>Honey शहद<br>Janam Ghutti जन्म घुट्टी<br>Others (Specify) अन्य (उल्लेख करें)	A<br>B<br>C<br>D<br>E<br>F<br>G<br>H<br>I<br>J<br>X

**421A**	What is the age of this child [CHILD NAME]?<br>(बच्चे का नाम) की उम्र क्या है?	Month महिना ___________		
**421B**	What is the sex of this child [CHILD NAME] ?<br>**बच्चे का लिंग क्या है?**	Male **पुरुष लिंग**<br>Female **महिला लिंग**		
**422**	Are you still breastfeeding [CHILD NAME]?<br>क्या आप (बच्चे का नाम) को अभी भी अपना दूध पिला रही है?	Yes हां<br>No नहीं	1<br>2	 ►424<br>**यदि प्रश्न संख्या 422 में 2 कोड हुआ है तो प्रश्न संख्या 424 पर**

				जाएँ
**423**	How many times did you breastfeed [CHILD NAME] in the last 24 hours?<br>पिछले 24 घंटों में आपने (बच्चे का नाम) को कितनी बार अपना दूध पिलाया ?	संख्या<br>Number__________		
**424**	For how many months did you breastfeed [CHILD NAME]? आपने (बच्चे का नाम) कितने महीनो तक अपना दूध पिलाया था	Months महीने<br>______________		
**425**	For how many months did you exclusively breastfeed that is no other food or liquid was given to this child?<br>आपने (बच्चे का नाम) कितने समय तक केवल अपना दूध पिलाया था यानि उसके अलावा कोई और खाने व पीने की चीज नहीं दी ?	Months महीने<br>______________		
	See Q422. If in 422, answer is 1, then goto 426.			
**425a**	Why did you stop breastfeeding the child?<br><br>आपने (बच्चे का नाम) को दूध पिलाना क्यों बन्द कर दिया था ?<br><br>**PROBE:** Any others?<br>कुछ और<br><br>**CODE ALL THAT APPLY**<br>**जो लागू हो सभी को कोड करें**	Problem with breast (pain, cracked nipples, engorgement) (A)....................................<br>स्तनों में परेशानी (दर्द दरार वाले निप्पल ठूंसे निप्पल)<br>Child did not suck well (B)....................................<br>बच्चा सही प्रकार से चूस नहीं पाता था<br>Not enough time to feed (c) ....<br>दूध पिलाने के लिये पर्याप्त समय नहीं था<br>Mother felt not enough breast milk (D)..............<br>मॉ को लगा कि स्तनों में पर्याप्त दूध नहीं है<br>Mother got pregnant/ new infant born (E).......<br>मॉ गर्भवती हो गयी थी/नवजात बच्च पैदा हुआ था<br>Mother went back to work (F)....................................<br>मॉ काम पर वापिस चली गयी थी<br>Infant resisted breastfeeding (G) ....<br>नवजात मॉ का दूध पीने का विरोध कर रहा था<br>Infant already grown up (H) ....<br>नवजात बडा हो गया था<br>OTHER (SPECIFY) अन्य (स्पष्ट करें) (X) ....<br>_______		
**426**	During the time that you exclusively breastfed, did you give water to the baby?<br><br>जब तक आपने (बच्चे का नाम) को अपना दूध पिलाया क्या	Yes हां<br>No नहीं	1<br>2	

	आपने उसे पीने के लिए पानी दिया था ?			

**427**	Now I would like to ask you about the liquids [CHILD NAME] drank yesterday during the day or at night.<br>अब मैं यह जानना चाहूंगी कि (बच्चे का नाम) ने कल दिन और रात मिलाकर कौन कौन सी चीजें पी ?<br><br>**READ (a-h) BELOW AND CODE YES OR NO FOR EACH.**<br><br>**प्रत्येक के बारे में अलग अलग पूछें**			If child is younger than 5 months and 30 days and option in 427="no"<br>यदि बच्चा 5 महीने 30 दिन से कम आयु का है और जी19 का उत्तर=''नहीं'' है तो<br>"Have you ever given [liquid] at any time to [CHILDNAME]"?<br>क्या आपने कभी भी (बच्चे का नाम) को तरल पदार्थ पिलाया है?	
	If 427A_1 =2 skip to 427A_2;				
	**Liquids**	**Yes**<br>**हाँ**	**No**<br>**नहीं**	**Yes**<br>**हाँ**	**No**<br>**नहीं**
	a. Plain water? सादा पानी	1	2	1	2
	b. Commercially produced infant formula milk? बाजार से मिलने वाला नवजात का फार्मूले वाला दूध	1	2	1	2
	c. Any other kind of milk (tinned, powdered, or fresh animal milk)? किसी अन्य प्रकार का दूध (टिन वाला पाउडर वाला या ताजा जानवर का दूध)	1	2	1	2
	d. Fruit juice? फल का जूस	1	2	1	2
	e. Tea or coffee? चाय/काफी	1	2	1	2
	f. Sodas like Pepsi, Coke, Orange drink? सोडा जैसे पेप्सी कोक, ऑरेन्ज पेय	1	2	1	2
	g. Clear broth/rice water/soup/boiled water? सोडा षोरबा/चावल का पानी/सूप/उबला हुआ पानी	1	2	1	2
	X. Other liquids (SPECIFY) अन्य तरल पदार्थ ;उल्लेख करें	1	2	1	2

**428**	Does this child eat any solid, semi-solid or soft foods?<br>क्या (बच्चे का नाम) कोई मसला हुआ, मुलायम या ठोस खाना खाता है ?	Yes हां<br>No नहीं	1<br>2	►431<br>यदि प्रश्न संख्या 428 में 2 कोड हुआ है तो प्रश्न संख्या 431 पर जाएँ
**429**	When did this child begin eating semi-solid, soft foods?<br>(बच्चे का नाम) ने ऐसा ठोस मसला हुआ सा मुलायम भोजन खाना किस आयु से शुरू किया था ?	Age in completed Months<br>उम्र पूर्ण महीनों में	______	
**430**	When the child falls sick, how regularly do you feed your child semi-solid or solid food in comparison to the child's normal	Same as before कोई अंतर नहीं	1	
		Less number of times than before पहले से कम बार देती हैं	2	
		More than before पहले से ज्यादा देती हैं	3	

	intake?<br>जब बच्चा बीमार होता है तो आप उसे तबीयत ठीक होने की तुलना में कितनी बार मसला हुआ मुलायम या ठोस खाना देती हैं?<br><br>**एक ही उत्तर की अपेक्षा**<br>**SINGLE CODING ONLY**	Do not give semi-solid or solid food during sickness<br>तबीयत खराब होने पर कोई मसला हुआ मुलायम या ठोस खाना नहीं देती है	4	
		Child did not fall sick कह नहीं सकती क्योंकि बच्चा कभी बीमार नहीं हुआ	9	
**431**	After the delivery, did a health worker such as an ANM, ASHA, or Anganwadi Worker come to your home to check on you and your baby? प्रसव के बाद क्या स्वास्थ्य कार्यकर्ता जैसे एएनएम, आषा या आंगनवाडी कार्यकर्ता आपको या नवजात षिषु को देखने आपके घर आये थे?	Yes हां<br>No नहीं	1<br>2	►435<br>यदि प्रश्न संख्या 431 में 2 कोड हुआ है तो प्रश्न संख्या 435 पर जाएँ
**432**	Who came to visit you and the baby?<br>आपको और आपके बच्चे को देखने कौन आया था? **क्या ये** आषा, आंगनवाडी कार्यकर्ता या एएनएम में से थे<br><br>**एक से ज्यादा जवाब संभव हैं**	ASHA आषा<br>ANM एएनएम<br>Aaganwadi worker आंगनवाडी कार्यकर्ता<br>Other (Specify) अन्य कोई (उल्लेख करें)	A<br>B<br>C<br>X	
**433**	How many days or weeks after the delivery did the first home visit take place?<br>प्रसव के बाद, कितने दिनों या सप्ताह के बाद पहली बार देखने के लिए आपके घर आये	Days<br>थ्दन<br>Weeks	_____	

**434**	How many times did an ASHA, AWW, ANM visit you at home during the <u>first week</u> after you gave birth to your child?<br>नवजात षिषु को जन्म देने के बाद पहले सप्ताह में कितनी बार आषा, आंगनवाडी कार्यकर्ता या एएनएम आपके घर आये?	Number of times<br>कितनी बार		
**435**	After the delivery, did any community group member come to your home to check you and your baby?<br>प्रसव के बाद क्या सामुदायिक समूह के सदस्य आपको या नवजात षिषु को देखने आपके घर आये थे?	Yes हां<br>No नहीं<br>There was no group at that time<br>उस समय कोई समूह नहीं था<br>Respondent was not member of the group at that time<br>उस समय उत्तरदाता समूह की सदस्य नहीं थी	1<br>2<br>3<br>4	►502<br>►502<br>►502<br>यदि प्रश्न संख्या 435 में 1 से ऊपर

				कोड हुआ है तो प्रश्न संख्या 502 पर जाएँ
**436**	How many days or weeks after the delivery did the first home visit by such community members take place?<br>प्रसव के बाद, कितने दिनों या सप्ताह के बाद **समूह के सदस्य** पहली बार **आपको और बच्चे को** देखने के लिए आपके घर आये?	Days		

**437**	Did the child [CHILD NAME] suffer from :<br>क्या बच्चे को कभी निम्न बीमारियाँ हुई हैं?<br>Response categories : उत्तर श्रेणी<br>1 = Yes हाँ<br>2 = No नहीं<br><br>The type of doctor from where received treatment<br>किस प्रकार के डॉक्टर से इलाज कराया?<br><br>A) Doctor in PHC or sub-center - प्राथमिक स्वास्थय केंद्र या उप स्वास्थय केंद्र के डॉक्टर से<br>B) District hospital<br>जिला अस्पताल से<br>C) City doctor through Sky clinic<br>स्काई क्लिनिक के डॉक्टर से<br>D) Local private doctor निजी/प्राइवेट डॉक्टर से<br>E) Local jhola chaap - झोला झाप डॉक्टर से		Ever suffered कभी पीड़ित हुआ?<br>(A)	Currently suffering इस समय पीडित है?<br>(B)	Did the child recieve treatment ?<br>बच्चे का इलाज हुआ?<br>(C)	From whom ?<br>किस से इलाज हुआ?<br>(D)
		Diarrohea (with fever, lethargy, persistent vomitting and blood in stool) दारिया (बुखार के साथ सुस्ती, लगातार उल्टियां, और टट्टी में खून)	1 हाँ<br>2 नहीं	1 हाँ<br>2 नहीं	1 हाँ<br>2 नहीं	A B C D E
		Pneumonia (Ill with rapid short breathing, fever & cough) निमोनिया (सास का	1 हाँ<br>2 नहीं	1 हाँ<br>2 नहीं	1 हाँ<br>2 नहीं	A B C D E

		फूलना,बुखार, बलगम)				
		Kala azar<br>काला अज़र	1 हाँ<br>2 नहीं	1 हाँ<br>2 नहीं	1 हाँ<br>2 नहीं	A B C D E

BLOCK 5: IMMUNIZATION

#	Question	Answers	Codes	Skip to
502	Did [CHILD NAME] ever receive any vaccinations to prevent (him/her) from getting diseases, including vaccinations received in a Pulse Polio program?<br>क्या (बच्चे का नाम) को कभी बिमारियो से बचाव के लिये कोई टीका लगा है इसमें पल्स पोलीयो कार्यक्रम में दिए गए टीकों को भी शामिल करें।	Yes हां<br>No नहीं	1<br>2	 ►601<br>यदि प्रश्न संख्या 502 में 2 कोड हुआ है तो प्रश्न संख्या 601 पर जाएँ
503	Do you have a card where vaccination details are written down?<br>क्या आपके पास **जच्चा-बच्चा** कार्ड है जिस पर (बच्चे का नाम) के सभी टीके लिखे व दर्ज किए गए हैं ?<br><br>(IF YES, MAY I SEE IT, PLEASE?)<br>यदि हॉ क्या मै कार्ड देख सकता हूँ	Yes, Seen हॉ, मैने देखा<br>Yes, Not seen हॉ, मैने नही देखा<br>No Card कार्ड नहीं	01<br>02<br>03	 ►506<br>►506<br>यदि प्रश्न संख्या 503 में 1 से ऊपर कोड हुआ है तो प्रश्न संख्या 506 पर जाएँ

			**Yes**<br>**हाँ**	**No**<br>**नहीं**	
504	Investigator to record from the card all the vaccinations child received?<br>अन्वेषक कार्ड से दर्ज कर लिखें कि बच्चे ने कौन कौन सा टीका लगवाया है?				
		BCG बीसीजी	1	2	
		P0 [Polio given at birth] पोलीयो 0 पोलीयो जन्म के समय दिया गया	1	2	
		Polio पोलीयो 1	1	2	
		DPT डीपीटी 1	1	2	
		Polio पोलीयो 2	1	2	
		DPT डीपीटी 2	1	2	
		Polio पोलीयो 3	1	2	
		DPT डीपीटी 3	1	2	

		Measles खसरा	1	2	
		Vitamin A [First dose]<br>विटामिन ए(पहली खुराक)	1	2	
505	Has this child received any vaccinations that are not recorded on this card?<br>बच्चे ने ऐसा कोई टीका लगवाया है जो कि इस कार्ड पर दर्ज नहीं है<br><br>यदि उत्तरदाता "हाँ" कहती है तो उससे/ या उसके परिवार के किसी सदस्य से, टीके के बारे में जानकारी लेने की कोशिश करें| इस प्रश्न में "हाँ" तभी दर्ज करें जब बच्चे को निन्मलिखित में से कोई टीका लगा है (जिसका विवरण कार्ड में दर्ज नहीं हुआ है):<br>**बीसीजी, पोलियो 0-3, डीपीटी 1-3, खसरा**<br>**यदि इस प्रश्न संख्या में "हाँ" कोड होता है है तो, वापस प्रश्न्संख्या 505 में जाएँ, और लगाये गए टीकों को कोड करें|**	Yes हां<br>No नहीं	1<br>2		►601 यदि प्रश्न संख्या 505 में 2 कोड हुआ है तो प्रश्न संख्या 601 पर जाएँ

<u>Please tell me, did the child receive:</u> <u>कृपया मुझे बताएं कि क्या बच्चे को निम्नलिखित टीका लगा है?</u>

506	A BCG vaccination against tuberculosis, that is, an injection in the arm or shoulder that usually causes a scar?<br>बीसीजी का टीका जो टीबी से लडने के लिये दिया जाता है और हाथ या कंधे पर लगाया जाता और उसके लगने से निषान रह जाता है?	Yes हां<br>No नहीं	1<br>2	
507	Any POLIO VACCINE, that is, drops in the mouth, including vaccine received in a Pulse Polio campaign?<br>पोलीयो टीका	Yes हां<br>No नहीं	1<br>2	
508	Was the first POLIO VACCINE received in the first two weeks after birth or later?<br>क्या जन्म के दो सप्ताह के बीच बच्चे को पोलियो का पहला लगा था ?	Yes हां<br>No नहीं	1<br>2	► 510 यदि प्रश्न संख्या 508 में 2 कोड हुआ है तो प्रश्न संख्या 510 पर जाएँ
509	How many times was the Polio vaccine given?<br>पोलीयो का टीका कितनी बार लगा?<br>(excluding Polio '0' and pulse polio)<br>(पोलीयो '0' और पल्स पोलीयो को छोडकर)	Number<br>संख्या	_____	
510	A DPT vaccination, that is, and injection given in the thigh or buttocks, sometimes at the same time as polio drops?<br>डीपीटी का टीका जो की जांघ या कूल्हो पर दिया जाता	Yes हां<br><br>No नहीं	1<br><br>2	 <br>► 512 यदि प्रश्न

	है और कई बार यह पोलियो की दवाई के साथ भी दिया जाता है?			संख्या 510 में 2 कोड हुआ है तो प्रश्न संख्या 512 पर जाएँ
511	How many times was a DPT vaccination received?<br>डीपीटी का टीका कितनी बार लगा?	Number<br>संख्या	_____	
512	Was an injection against MEASLES given at right arm/shoulder?<br>खसरे से बचाव का टीका	Yes हां<br>No नहीं	1<br>2	
513	The first dose of Vitamin A?<br>विटामिन A की पहली खुराक?	Yes हां<br>No नहीं	1<br>2	

BLOCK 6: FAMILY PLANNING

अगले कुछ प्रष्न परिवार नियोजन या अलग–अलग विधि के बारे में है जो एक दम्पति गर्भधारण को रोकने या देर करने के लिये प्रयोग कर सकते है

601	Which ways or methods have you heard about?<br>**कौन कौन सी विधियों के बारें में आपने सुना हैं?**<br><br>FOR METHODS NOT MENTIONED SPONTANEOUSLY, ASK: **जो विधियां खुद से ना बतातिएँ, उनके लिए पूछें:**<br>Have you ever heard of (METHOD)?<br>क्या आपने कभी (विधि) का नाम सुना है?		602. Have you ever used [METHOD]?<br>क्या आपने कभी (विधि) प्रयोग किया है
a.	FEMALE STERILIZATION Women can have an operation to avoid having any more children.<br>महिला **नलबंदी** जिसमें एक महिला **को** और बच्चे न हो इसके लिये आपरेशन करा सकती है ?	Yes हां ....1<br>No नहीं 2	Yes हां ....1<br>No नहीं 2
b.	MALE STERILIZATION Men can have an operation to avoid having any more children.<br>**पुरूष नसबन्दी** एक पुरूष और बच्चे न हो इसके लिये आपरेषन करा सकता है ?	Yes हां ....1<br>No नहीं 2	Yes हां ....1<br>No नहीं 2
	If 602a=1 or 602b=1 skip to 605		
c.	PILL Women can take a pill every day or every week to avoid becoming pregnant.<br>**गर्भनिरोधक गोलियां** जो एक महिला गर्भवती होने से बचने के लिये प्रतिदिन या सप्ताह में एक बार खा सकती हैं	Yes हां ....1<br>No नहीं 2	Yes हां ....1<br>No नहीं 2
d.	IUD OR LOOP or Copper T Women can have a loop or coil placed inside them by a doctor or a nurse.<br>**आई यू डी या लूप या कॉपर टी** एक महिला डॉक्टर या नर्स द्वारा अपने गर्भाषय के अन्दर लगवा सकती है।	Yes हां ....1<br>No नहीं 2	Yes हां ....1<br>No नहीं 2

e.	INJECTABLES Women can have an injection by a health provider that stops them from becoming pregnant for one or more months.<br>**इन्जेक्शन** एक महिला किसी स्वास्थ्य प्रदाता से इन्जेक्षन लगवा सकती है जिससे वह एक महिने या ज्यादा महीने तक गर्भवती होने से बच सकती है।	Yes हां ....1<br>No नहीं 2	Yes हां ....1<br>No नहीं 2
f.	CONDOM OR NIRODH Men can put a rubber sheath on their penis before sexual intercourse.<br>**कान्डोम या निरोध** एक पुरूष यौन सम्बन्ध करने से पहले अपने लिंग पर एक रबर का आवरण चढा सकता है ।	Yes हां ....1<br>No नहीं 2	Yes हां ....1<br>No नहीं 2
g.	FEMALE CONDOM Women can place a sheath in their vagina before sexual intercourse.<br>**महिला कान्डोम** एक महिला यौन सम्बन्ध करने से पहले अपनी योनि में एक रबर का आवरण लगा सकती है ।	Yes हां ....1<br>No नहीं 2	Yes हां ....1<br>No नहीं 2
h.	RHYTHM METHOD Every month that a woman is sexually active she can avoid pregnancy by not having sexual intercourse on the days of the month she is most likely to get pregnant.<br>**सुरक्षित कालअवधि** प्रत्येक महीने एक महिला उन दिनों में यौन सम्बन्ध नहीं बना कर गर्भधारण से बच सकती है जब उसके गर्भधारण की संभावनाएं सबसे ज्यादा होती है।	Yes हां ....1<br>No नहीं 2	Yes हां ....1<br>No नहीं 2
i.	WITHDRAWAL Men can be careful and pull out before climax.<br>**बाहर निकालना** पुरूष सावधानी कर चरमावस्था पर पहुंचने से पहले अपना लिंग बाहर निकाल लेता है	Yes हां ....1<br>No नहीं 2	Yes हां ....1<br>No नहीं 2
j.	EMERGENCY CONTRACEPTION Women can take pills up to three days after sexual intercourse to avoid becoming pregnant.<br>**इमरजेन्सी गर्भनिरोधक** एक महिला यौन सम्बन्ध बनाने के तीन दिनों के अन्दर गोली ले गर्भधारण से बच सकती है	Yes हां ....1<br>No नहीं 2	Yes हां ....1<br>No नहीं 2
k.	Have you heard of any other ways or methods that women or men can use to avoid pregnancy?<br>क्या आपने किसी अन्य उपाय और विधियों के बारे में सुना है जो एक महिला या पुरुष गर्भधारण होने से बचने के लिये प्रयोग कर सकते है	Yes हां ....1<br>No नहीं 2	Yes हां ....1<br>No नहीं 2
603	Are you currently doing something or using any method to delay or avoid getting pregnant?<br>क्या आप वर्तमान में गर्भधारण को रोकने या उसमें देरी करने के लिए किसी विधि का प्रयोग कर रहे है ?	Yes हां ....1<br>No नहीं.............2 ► 701 **यदि प्रश्न संख्या 603 में 2 कोड हुआ है तो प्रश्न संख्या 701 पर जाएँ**	

604	Which method are you using?<br>आप कौन सी विधि का प्रयोग कर रहे है ?<br><br>एक से ज्यादा उत्तर संभव हैं	Female Sterilization महिला नसबंदी A<br><br>Male Sterilization रुष नसबंदी B<br><br>IUD or Loop आई यू डी या लूप C<br><br>Injectables इंजेक्शन D<br><br>Birth Control Pills गर्भनिरोधक प्रत्यारोपण E<br><br>Male Condoms कंडोम या निरोध F<br>Female Condoms महिला कंडोम G<br>Diaphragm गर्भनिरोधक टोपी H<br>Foam/jelly फोम/जैली I<br>Rhythm method सुरक्षित कालाविधि J<br>Withdrawal बाहर निकलना K<br>Emergency contraception इमरजेंसी गर्भनिरोधक L<br>Others (please specify) अन्य ;उल्लेख करेंद्ध X		
605	Was the current child [CHILD NAME] wanted then/later/never?<br>इस बच्चे को क्या आप पैदा करना चाहते थे (जब हुआ तब/बाद में/कभी नहीं?	Then उस वक्त<br>Later बाद में<br>Never कभी नहीं	1<br>2<br>3	
606	Now, I have some questions about the future.<br>Would you like to have (a/another) child, or would you prefer not to have any (more) children?<br><br>अब मैं आपसे भविष्य के बारे में कुछ प्रश्न पूछना चाहूंगी। क्या आप अभी एकऔर बच्चे चाहेंगे या कोई और बच्चा नहीं चाहेंगे ?	Prefer another child…..1<br>और बच्चा चाहेंगे<br>Prefer not to have another child…2<br>और बच्चा नहीं होना पसन्द करेंगे<br>Don't know/cant get pregnant…..3<br>पता नहीं/गर्भधारण नहीं कर सकते	1<br>2<br>3<br>4	
606b	Whose decision will it be to have or not have another child?<br><br>और बच्चे करने हैं या नहीं, यह किसका निर्णय होगा?	Yours आपका<br>Husband's आपके पति का<br>Joint decision दोनों<br>Mother-in-lawका सास का<br>Others अन्य	1<br>2<br>3<br>4<br>5	

607	Did you and your husband ever discuss about the following in the past one year?<br><br>क्या आपने और आपके पति ने निम्न विशिओं पर पिछले एक साल में बात की है?<br><br>Response categories: उत्तर श्रेणी<br>1 = Yes हाँ<br>2 = No नहीं	How many children to have कितने बच्चे करने हैं?<br>When to have children बच्चे कब करने हैं?<br>Which contraceptive method to use कौन सी गर्भ निरोधक विधि अपनानी चाहिए?<br>When to go for antenatal check-up - गर्भावस्था में प्रसव पूर्व चेकुप (जांच) के लिए कब जाना चाहिए?<br>Where to deliver a baby प्रसव कहाँ करना चाहिए?<br>How to maintain good hygiene स्वस्थ्य के लिए साफ़ सफाई कैसे रखनी चाहिए?<br>What to do for sanitation? घर में साफ़ सफाई के लिए क्या करना चाहिए?	1 2<br>1 2<br>1 2<br>1 2<br>1 2<br>1 2<br>1 2	

BLOCK 7: WATER, HYGEINE AND SANITATION

अब मैं आपसे पीने के पानी के स्रोतो और स्वच्छता/साफसफाई के बारे में आपसे कुछ प्रश्न पुछना चाहूंगी

#	Question	Answers	Codes	Skip to
701	Has any health worker, ASHA, AWW or ANM, ever demonstrated proper hand washing to you?<br>क्या किसी स्वास्थ्य कार्यकर्ता जैसे कि आशा, आंगनवाडी कायकर्ता या एएनएम ने आपको सही तरीके से हाथ कैसे धोते हैं ऐसा दिखलाया है ?	Yes हां<br>No नहीं	1<br>2	
701 b	Has your Saheli ever demonstrated proper hand washing to you?<br>क्या कभी सहेली ने आपको सही तरीके से हाथ कैसे धोते है दिखलाया है६	Yes<br>No	1<br>2	
702	According to you, what are the occasions before which one should always wash hands with soap?<br>आपके अनुसार, कौन से अवसरों से पहले आपको साबुन से हमेशा हाथ **अवश्य** धोना चाहिए?<br><br>**PROBE: Anyone else?**<br>**और कोई?**<br>**एक से ज्यादा उत्तर संभव हैं**	Before cooking खाना बनाने से पहले<br>Before touching or eating खाने या छूने से पहले<br>Before feeding someone किसी को खिलाने से पहले<br>Before Praying प्रार्थना से पहले<br>Others (specify) अन्य (उल्लेख करें)<br>Don't know पता नहीं<br>None कोई भी नहीं	A<br>B<br>C<br>D<br>X<br>Y<br>Z	

703	According to you, what are the occasions after which one should always wash hands with soap?<br>आपके अनुसार, कौन से अवसरों के बाद आपको साबुन से हमेशा हाथ धोना चाहिए?<br><br>**PROBE: Anyone else?**<br>**और कोई?**<br><br>**एक से ज्यादा उत्तर संभव हैं**	After defecation शौच के बाद<br>After urinating पेशाब करने के बाद<br>After eating खाने के बाद<br>After sweeping/mopping/dusting साफ सफाई/झाडू पोछा करने के बाद<br>After touching pets or handling animals and their waste पालतु जानवरों को छुने या उनकी गोबर/गंदगी निकालने के बाद<br>After blowing nose orcoughing बही हुई नाक साफ करने के बाद<br>After cleaning utensils बर्तन साफ करने के बाद<br>After cleaning a child's bottom बच्चे के नीचे भाग के सफाई के बाद<br>After working in the field खेत में काम करने के बाद<br>After playing खेलने के बाद<br>Others (specify) अन्य (उल्लेख करें)<br>None कोई भी नहीं	A<br>B<br>C<br>D<br>E<br><br>F<br>G<br>H<br>I<br>J<br>X<br>Y

704	In general, do you wash your hands with soap during a typical day?<br>सामान्य रूप में, क्या आप अपना हाथ दिन में **साबुन से** धोती है?	Yes हां<br>No नहीं	1<br>2	 **► 706**<br>**यदि प्रश्न संख्या 704 में 2 कोड हुआ है तो प्रश्न संख्या 706 पर जाएँ**
705	When do you usually wash <u>your</u> hands with soap and water during a typical day?<br>सामान्यता एक दिन में आप कब–कब अपने हाथ **साबुन और पानी से** धोते हैं ?<br><br>**PROBE: Anyone else?**<br>**और कोई?**<br><br>**एक से ज्यादा उत्तर संभव हैं**	Before cooking खाना बनाने से पहले<br>After using the toilet शौचालय का उपयोग करने के बाद<br>After handling food भोजन से निपटने के बाद<br>Before eating खाने से पहले<br>Before praying प्रार्थना से पहले<br>Before feeding the infant षिषु को खिलाने के पहले<br>After cleaning infant feces शिषु मल की सफाई करने के बाद<br>After dusting/ sweeping/ mopping साफ सफाई/झाडू पोछा करने के बाद<br>After touching pets or handling with animals and their waste पालतु जानवरों को छुने या उनकी गोबर/गंदगी निकालने के बाद<br>After blowing nose or coughing बही हुई नाक साफ करने के बाद<br>After cleaning utensils बर्तन साफ करने के बाद<br>Other (specify) अन्य (उल्लेख करें)	A<br>B<br>C<br>D<br>E<br>F<br>G<br>H<br><br>I<br><br>J<br>K<br>*X*	
706	How do you wash your hands after using the toilet?<br>शौचालय का उपयोग करने के बाद आप अपने हाथो को ज्यादातर कैसे धोती है?	Using only water केवल पानी का उपयोग<br>Using soap साबून का उपयोग<br>Using mud/ soil किचड/मिट्टी का उपयोग<br>Using ash राख का उपयोग	1<br>2<br>3<br>4	

706a	The last time you used the toilet, did you wash your hands afterwards<br><br>पिछली बार जब आपने शौचालय का उपयोग किया तो क्या उसके बाद अपने हाथ धोये थे?	Yes…हाँ ………1<br>No…नहीं………..2		► **707**<br>यदि प्रश्न संख्या 706a में 2 कोड हुआ है तो प्रश्न संख्या 706c पर जाएँ
706 b	If yes, what did you use (water only/water and soap/water and surf/ash/oil/other)<br><br>यदि हां तो आपने हाथ धोने के लिए किसका उपयोग किया (केवल पानी/पानी और साबुन/पानी और सर्फ/राख/मिट्‌टी/अन्य)?<br><br>**CODE ONE ONLY**<br>सिर्फ एक उत्तर कोड करे	Water only………केवल पानी………………………1<br>Water and soap…पानी और साबुन……………………….2<br>Water and surf…पानी और सर्फ………………………..3<br>Ash………………राख……………………...4<br>Soil………………मिटटी ………………………5<br>Other (specify)… …अन्य …………………….6		
706c	Yesterday, after cleaning the child (after his/her stool) did you wash your hands with soap?<br><br>**बच्चे की शौच साफ़ करने के बाद क्या आपने कल अपने हाथ को साबुन से धोया था?**			
706 d	Is soap available in the household?<br>क्या घर में साबुन उपलब्ध है?	Yes……हाँ…………………………………1<br>No……नहीं………………………………..2		

707	The last time [CHILD NAME] passed stools, what was done to dispose of the stools?<br>अन्तिम बार जब (बच्चे का नाम) ने मल/पखाना किया था तब मल को कहां फेंका गया था ?	Child used toilet or latrine बच्चे ने टायलेट प्रयोग किया था<br>Put/ rinsed into toilet or latrine टायलेट या लेटरिन में धोया था<br>Put/ rinsed into drain or ditch नाली या नल पर धोया था<br>Thrown into garbage कूडे में डाल दिया था<br>Buried जला दिया था<br>Left/ washed in the open खुले में डाल दिया/धो दिया था<br>Other अन्य	1<br>2<br>3<br>4<br>5<br>6<br>7	
708	What is the main source of drinking water for members of your household?<br>आपके परिवार के सदस्यों के लिये पीने के पानी का मुख्य स्रोत क्या है	Piped into dwelling घर में पाइप (नल)<br>Piped to yard/plot आंगन/प्लाट का पाइप (नल)<br>Public tap/ standpipe सार्वजानिक नल<br>Tube well or borehole टयूबवेल/बोरहोल/ चापाकल<br>Protected well ढका कुआ<br>Unprotected well खुला कुआ<br>Water from spring मौसमी पानी<br>Protected spring सुरक्षित मौसमी पानी<br>Unprotected Spring असुरक्षित मौसमी पानी<br>Rainwater बरसात का पानी<br>Tanker truck टैंकर ट्रक<br>Cart with small tank छोटे टैंक वाली गाडी<br>Surface water (river/ dam/ lake/ pond/ stream/ canal/ irrigation channel) सतही पानी (नदी/बांध/झील/तालाब /धारा/नहर/सिंचाई चैनल)<br>Bottled water बोतल का पानी	1<br>2<br>3<br>4<br>5<br>6<br>7<br>8<br>9<br>10<br>11<br>12<br>13<br>14	► 712<br>यदि प्रश्न संख्या 708 में 1 कोड हुआ है तो प्रश्न संख्या 712 पर जाएँ
709	Where is the water source located?<br>पानी का स्रोत कहां पर स्थिति है?	In own yard/plot खुद के यार्ड/जमीन पर<br>Elsewhere अन्यत्र	1<br>2	► 712<br>यदि प्रश्न संख्या 709 में 1 कोड हुआ है तो प्रश्न संख्या 712 पर जाएँ
710	How long does it take to go there, get water, and come back in one trip?<br>एक बार में वहां जाकर पानी लेने और पानी लेकर आने में कितना समय लगता है?	In Minutes मिनिटों में	__ __ __	
711	Who usually goes to this source to fetch water?<br>आमतौर पर पानी लाने उस जगह पर कौन जाता है?	Adult woman वयस्क महिला<br>Adult man वयस्क पुरूष<br>Female child (under 15years) महिला बच्चे (15 वर्ष से कम)<br>Male child (under 15years) पुरूष बच्चे (15 वर्ष से कम)	1<br>2<br>3<br>4	

712	Do you store water for future use (for use beyond the day water is collected)?<br>भविष्य में उपयोग के लिए क्या आप पीने के पानी को सुरक्षित रखते है?	Yes हां<br>No नहीं	1<br>2	 ► 715 यदि प्रश्न संख्या 712 में 2 कोड हुआ है तो प्रश्न संख्या 715 पर जाएँ
713	For how long do you store drinking water for future use?<br>कितने समय के लिए आप पीने के पानी को भविष्य में उपयोग के लिए सुरक्षित रखते है?	One week एक सप्ताह के लिए<br>Two weeks दो सप्ताह के लिए<br>One month एक महीने के लिए<br>More than one month एक महीने से ज्यादा के लिए	1<br>2<br>3<br>4	
714	How do you store drinking water?<br>आप पीने के पानी को संग्रह करके कहॉ रखते है?	Stored in covered container ढंके बरतन में संग्रह करके<br>Stored in open Container खुले बरतन में संग्रह करके	1<br>2	
715	Do you treat your water in any way to make it safer to drink?<br>क्या आप पीने के पानी को किसी भी प्रकार से सुरक्षित बनाती हैं ?	Yes हां<br>No नहीं	1<br>2	 ► 717 यदि प्रश्न संख्या 715 में 2 कोड हुआ है तो प्रश्न संख्या 717 पर जाएँ
716	What do you do to the water to make it safer to drink?<br>सामान्यतया पानी को पीने योग्य बनाने के लिये आप क्या करते है<br>**पूछें: और कोई?**<br>**उत्तरदाता द्वारा बताये गए जवाबों को क्रमवार तरीके से दर्ज करें। जैसे कि अगर महिला ज्यादातर बार फिटकिरी का प्रयोग करती है और कभी कभी पानी उबालती है तो पहले "B" दर्ज करें फिर "A" दर्ज करें**	Boil उबालना<br>Use Alum फिटकिरी का उपयोग<br>Add bleach/ chlorine tablets ब्लीच/क्लोरीन की गोलियां को मिलाना<br>Strain through a cloth कपडे के माध्यम से छानना<br>Use water filter (ceramic/ sand/ composite/ etc.) पानी के फिल्टर का इस्तेमाल करना(सेरेमिक/बालू/कम्पोजिट)<br>Use electronic purifier बिजली के प्यूरिफायर का इस्तेमाल करना<br>Let it stand and settle पानी को ठहरे रहने देना और स्थिर होने देना<br>Other (specified) अन्य (उल्लेख करें)	A<br>B<br>C<br>D<br>E<br>F<br>G<br>X	

717	What kind of toilet facility do members of your household usually use?<br>किस प्रकार की शौचालय सुविधा का उपयोग आपका परिवार कर रहे है ?	Flush to piped sewer system Ikkbi lhoj flLVe ls Qy'k<br>Flush to septic tank lsfIVd VSad ls Qy'k<br>Flush to pit latrine xM<s okYks 'k©pky; ls Qy'k<br>Flush to somewhere else dgha v©j Qy'k<br>Flush, don't know where फलश,पता नही कहॅा<br>Ventilated improved pit (VIP)/ biogas latrine हवादार (बेहतर) गडढा/बायोगैस शौचालय<br>Pit latrine with slab पट्टीवाले गडढे वाले शौचालय<br>Pit latrine without slab/ open pit बिना पट्टीवाले गडढे वाले शौचालय/ खुला गडढा<br>Twin pit/ composting toilet जुडे हुये गडढे/ कम्पोजिट शौचालय<br>Dry toilet सूखी शौचालय<br>No facility/ uses open space or field कोइ सुविधा नही/खुले स्थान या खेत का इस्तेमाल<br>Other (specify) अन्य (उल्लेख करें)____________	1<br>2<br>3<br>4<br>5<br>6<br><br>7<br>8<br><br>9<br>10<br>11<br><br>12	► 721<br>यदि प्रश्न संख्या 717 में 11 कोड हुआ है तो प्रश्न संख्या 721 पर जाएँ
718	Do you share this toilet facility with other household members?<br>क्या अन्य परिवार भी शौचालय की सुविधा को इस्तेमाल करते हैं ?	Yes हां<br>No नहीं	1<br>2	► 720<br>यदि प्रश्न संख्या 718 में 2 कोड हुआ है तो प्रश्न संख्या 720 पर जाएँ
719	How many households use this toilet facility?<br>इस शौचालय का इस्तेमाल **कितने** परिवार **करतें** हैं	Number<br>संख्या		
720	In the past week, has anyone in your household including children defecated in the open- for example in the field or in the river?<br>पिछले सप्ताह में आपके घर का कोई सदस्य या बच्चा शौच के लिए खुले में गया जैसे कि खुले मैदान में या नदी के पास?	Yes हां<br>No नहीं	1<br>2	

721	Where do you dispose of your household waste?<br>आप अपने घर के कचरे को कैसे निपटाते है?	Outside the house घर के बाहर<br>In a public garbage dump सार्वजनिक कूड़ाघर में<br>In a drain नाले में<br>Garbage collecter collects garbage कूड़ा ले जाने वाला घर से ले जाता है<br>Any other (specify) अन्य (स्पष्ट करें)	1<br>2<br>3<br>4<br><br>5	
722	Is waste (e.g. heaps of rubbish with household and/or human waste piled up) a problem where you live?<br>क्या कचरा **(जैसे कूड़े का ढेर अथवा मानवअपशिष्ट)** एक समस्या है आप जहां रहते हैं	Yes हां<br>No नहीं	1<br>2	► 723<br>यदि प्रश्न संख्या 722 में 2 कोड हुआ है तो प्रश्न संख्या 723 पर जाएँ
722a	What kind of waste presents a problem in your residential area**?**<br><br>**किस तरह की गंदिगी आपके घर के आस पास एक समस्या है?**	Household waste (e.g. vegetable/fruits peals, rotting food, polythene carry bags) घर का कूड़ा कचरा<br>Human waste मानव अपशिष्ट<br>Animal waste (cow dung, waste from domestic animals such as dogs, cats, goats etc) जानवरों का अपशिष्ट (जैसे कुत्ते बिल्ली द्वारा की गयी गंदिगी)<br>Drainage निकासी (नाली का कूड़ा)<br>Other अन्य	A<br><br>B<br>C<br><br>D	
723	What are the present methods of waste water disposal?<br>बेकार जल निपटान के वर्तमान तरीके क्या है?	Pour it off outside the house घर के बाहर बहा देते हैं<br>Pour in a drain नाली में बहा देते हैं<br>Dispose off in a farm खेत में बहा देते हैं<br>Pour in a nearby pond नजदीक के तालाब में बहा देते हैं<br>Other अन्य (स्पष्ट करें)	1<br>2<br>3<br>4<br>5	

BLOCK 8: SOCIAL NORMS ABOUT MNCHS BEHAVIORS

अब मैं महिलाओं और बच्चों के स्वास्थय से सम्बंधित व्यहवार के बारे में कुछ बयान पढ़ूंगी| कृपया मुझे बताये की आप इन बयानों से कितनी सहमत या असहमत हैं|

		**Strongly agree**<br>**पूरी तरह सहमत**	**Agree**<br>सहमत	**Disagree**<br>असहमत	**Strongly Disagree**<br>**बिल्कुल सहमत नहीं**
**ANC CHECK-UP** प्रसव पूर्व देखभाल से सम्बंधित					
**801**	Most women in my community think that 3 ANC check-ups during pregnancy are necessary<br>मेरे समुदाय में ज्यादातर महिलाओं को लगता है कि गर्भावस्था के दौरान 3 प्रसवपूर्व जांच आवश्यक है	1	2	3	4

**802**	Most women in my community think that 2 TT intake during pregnancy is necessary<br>मेरे समुदाय में ज्यादातर महिलाओं को लगता है कि गर्भावस्था के दौरान 2 टीटी सेवन आवश्यक है	1	2	3	4
**803**	Most women in my community think that 90 IFA intake during pregnancy is necessary<br>मेरे समुदाय में ज्यादातर महिलाओं को लगता है कि गर्भावस्था के दौरान 90 आइएएफ सेवन आवश्यक ह	1	2	3	4
**Delivery Preparedness and Safe Delivery** **प्रसव के लिए की गयी तैयारिओं और सुरक्षित प्रसव के बारे में**					
**804**	Most pregnant women in my community do not prepare in advance for the delivery<br>मेरे समुदाय में ज्यादातर गर्भवती महिलाएं प्रसव के बारे में पहले से तैयारी नहीं करके रखती	1	2	3	4
**805**	If I arrange for a skilled birth attendant for conducting my home delivery, people around me will think I am being overcautious about my delivery.<br>यदि मैं मेरे प्रसव के लिए एक कुषल सहायक की व्यवस्था करती हूॅ तो मेरे आस पास के लोगो को लगेगा कि मैं कुछ ज्यादा हि परवाह कर रही हूॅ	1	2	3	4
**806**	Most pregnant women in my community deliver at home<br>मेरे समुदाय में ज्यादातर गर्भवती महिलाओं का प्रसव घर पर होता है	1	2	3	4
**807**	My mother-in-law will think I am a ‘difficult’ person if I ask to deliver at a facility.<br>मेरी सास को लगता है कि मै परेषान करने वाली महिला हूॅ अगर मैं प्रसव किसी स्वास्थ्य सुविधा पर कराने के लिए कहती हूॅ<br><br>**यदि सास की मृत्यु हो गयी है/ या सास साथ में नहीं रहती है तो “9” दर्ज करें**	1	2	3	4
PNC **प्रसव के बाद महिला और बच्चे की देखभाल**					
**808**	Most people in my community do not consider a post natal checkup (PNC) important.<br>मेरे समुदाय में ज्यादातर लोगों का ऐसा मानना है कि प्रसव के बाद देखभाल महत्वपूर्ण नहीं है	1	2	3	4
**809**	It is common for women in my community to get a complete set of PNC done.<br>मेरे समुदाय की महिलाओं में प्रसव के बाद पूरी देखभाल मिलना आम बात है	1	2	3	4
**810**	I fear the elder will not think well of me of me if I insist on getting a PNC check-up.<br>मुझे इस बात का डर है कि मेरे समुदाय के बड़े–बुजुर्गों मेरे बारे में बुरा सोचेंगे, अगर **मैं** प्रसव के बाद होने वाली जांच के लिए जोर देती हूॅ	1	2	3	4
Family Planning **परिवार नियोजन**					

**811**	**Birth** Spacing is considered to be a good thing among most people I know<br>मेरी जान–पहचान में ज्यादातर लोग बच्चों में अंतर रखने को अच्छा मानते हैं	1	2	3	4
**812**	Women of my age in my community will disapprove of me if they find out that we are using family planning methods.<br>मेरे समूह की महिलाएं मुझे पसंद नहीं करेंगी अगर उन्हें पता चलेगा कि मैं परिवार नियोजन के साधनों का प्रयोग करती हूँ	1	2	3	4
Newborn care (keeping baby warm, bathing) **नवजात शिशु की देखभाल**					
**813**	In my community, mother of newborn provides direct body warmth to the newborn.<br>मेरे समूह में महिलाऍं नवजात षिषु को कंगारू विधि से बच्चों को गर्म रखती हैं<br>**ध्यान दें: उत्तरदाता को** कंगारू विधि **के बारे में** बताए	1	2	3	4
**814**	People in my community usually apply something on the child's stump.<br>मेरे समुदाय में, आम तौर पर बच्चे के नाल या नाभि पर कुछ लगाते है	1	2	3	4
**815**	Newborns are bathed immediately after birth in my community.<br>मेरे समुदाय में, जन्म के तुरंत बाद बच्चे को नहलाते है	1	2	3	4
Breastfeeding **नवजात शिशु को माँ के दूध पिलाने से सम्बंधित**					
**816**	Most people in my community start breastfeeding within 1 hour of birth.<br>मेरे समुदाय में अधिकांष लोग जन्म के 1 घंटे के भीतर **बच्चे को** स्तनपान कराते **हैं**	1	2	3	4
**817**	Most newborns in my community are fed homemade mixtures like honey and "tulsi" (basil).<br>मेरे समुदाय में ज्यादातर नवजात षिषुओं को घर में बनाये गए मिश्रण जैसे कि शहद और तुलसी, या मिश्री और पानी पिलाया जाता है	1	2	3	4
**818**	A lot of women in my community feed their children other things along with breast milk before the age of 6 months.<br>मेरे समुदाय में महिलाएं अपने बच्चे को 6 महीने उम्र के पहले मॉ के दूध के अलावा अन्य खाने की चीजे खिलाती है	1	2	3	4
**819**	People in my community will think I am keeping the child hungry if I feed the child just breast milk up to the age of 6 months<br>मेरे समुदाय के लोग सोचते है कि मैं अपने बच्चे को भूखा रखती हूँ यदि मैं उसे छः महीने की उम्र तक अपने दूध के अलावा कुछ खाने की चीज नहीं देती	1	2	3	4
Age appropriate complementary feeding **बच्चों को बाहर से दिए जाने वाले आहार के बारे में**					

**820**	Most people in the community follow their mother-in-law's advice on feeding newborns and children<br>मेरे समुदाय में अधिकतर महिलाएं अपनी सास की सलाह पर अपने नवजात षिषु और बच्चों को खिलाती है	1	2	3	4
Immunization **बच्चों के टीकाकरण से सम्बंधित**					
**821**	People in my community will think of me as irresponsible if I do not take my child for immunization.<br>मेरे समुदाय के लोग मुझे गैर जिम्मेदार समझेंगे यदि में अपने बच्चे को टीकाकरण के लिए नहीं ले जाती	1	2	3	4
**822**	Most people in my community get their children fully immunized<br>मेरे समुदाय के अधिकांष लोग अपने बच्चे को बीमारियों से बचाव के सभी टीके लगवाते हैं	1	2	3	4
Sanitation **साफ़ सफाई से सम्बंधित**					
**823**	Most people in my community prefer to wash their hands with ash or mud<br>मेरे समुदाय में अधिकांष लोग अपना हाथ धोने के लिए राख या मिट्टी **को पसंद (प्राथमिकता) करतें** है	1	2	3	4
**824**	Most people in my community wash their hands only after daily ablutions<br>मेरे समुदाय के अधिकांष लोग सिर्फ दैनिक/नित्य कियाकर्म के बाद ही अपना हाथ धोते है	1	2	3	4
**825**	Most people in my community do not boil water before drinking<br>मेरे समुदाय में अधिकांष लोग पीने से पहले पानी नहीं उबालते हैं	1	2	3	4
**826**	Most people in my community store potable water in mud urns<br>मेरे समुदाय में अधिकांष लोग पीने के पानी को मिट्टी के बर्तन (घड़े/सुराही) में रखते है	1	2	3	4
**827**	Most people in my community perform daily ablutions in public spaces<br>मेरे समुदाय अधिकांष लोग दैनिक/नित्य कियाकर्म सार्वजनिक स्थानों पर करते हैं	1	2	3	4
**828**	Most people in the community would support community toilet facilities<br>समुदाय के अधिकांष लोग सामुदायिक शौचालय की सुविधा का समर्थन करेंगे	1	2	3	4
**829**	Most people in my community follow age old methods (such as throwing outside the house) of waste disposal<br>मेरे समुदाय में ज्यादातर लोग अपशिष्ट पदार्थ जैसे की घरेलु कूड़ा, गोबर, घर से निकलने वाला गन्दा पानी के निस्तारण के पुराने तरीको का पालन करते है	1	2	3	4

BLOCK 9: COMMUNITY MOBILIZATION QUESTIONS

The next set of questions is about the community groups that exist in your village. I would like to ask you some questions regarding your participation, access to community groups and their utility.
अगले प्रश्न आपके आपके गांव में मौजूद समुदायिक समुहों के बारे में हैं। मैं आपसे कुछ सवाल समूह में आपकी भागीदारी, सामुदायिक समूहों तक पहुँच, और उनकी उपयोगिता के बारे में पूछना चाहती हूँ

#	Question	Answers	Codes	Skip to
**901**	Since how long have you been member of the community group?<br>कब से आप समुदायिक समूह के सदस्य रहें हैं?	Number of months<br>महीनों की संख्या		
**902**	How many members are there in your group?<br>आपके समूह में कुल कितने सदस्य हैं?	Number of members<br>सदस्यों की संख्या		
**905**	How often does your group meet for discussions or activities?<br>कितनी बार आपका समूह चर्चाओं या गतिविधियों के बारे में मिलते हैं?	Weekly सप्ताह में एक बार<br>Bi-monthly महीने में दो बार<br>Monthly महिने में एक बार<br>Occasionally कभी कभी<br>Never met so far कभी नहीं मिले अब तक	1<br>2<br>3<br>4<br>5	► 911<br>यदि प्रश्न संख्या 905 में 5 कोड हुआ है तो प्रश्न संख्या 911 पर जाएँ
**906**	How often do you participate in the group meetings?<br>आप कब–कब समूह बैठक में भाग लेती हैं?	Weekly सप्ताह में एक बार<br>Bi-monthly महीने में दो बार<br>Monthly महिने में एक बार<br>Occasionally कभी कभी<br>Never कभी बैठक में भाग नहीं लिया	1<br>2<br>3<br>4<br>5	
**906 A**	When was the last time that you participated in a group meeting ?<br>आपने आखिरी बार समूह की मीटिंग/बैठक में कब भाग लिया था?	Days ____<br>दिन______		
**907**	Does your group ever discuss about any of the health topics that are concerned with pregnant women and young mothers?<br>क्या समूह में कभी भी स्वास्थ्य संबंधित विषयों पर चर्चा हुई जो गर्भवती महिलाओं या युवा मातओं से संबंधित हो?	Yes हां<br>No नहीं	1<br>2	► 909<br>यदि प्रश्न संख्या 907 में 2 कोड हुआ है तो प्रश्न संख्या 909 पर जाएँ
**908**	Which topics were discussed in a recent meeting?<br>पिछले समूह बैठक में किन मुद्दों पर चर्चा हुई?	What to do if a pregnant woman has an emergency A<br>गर्भवती महीला को अमरजेंसी/अचानक आई		

	एक से ज्यादा उत्तर संभव हैं	दिक्कतों मे क्या करना चाहिये<br>Antenatal care of mothers B<br>प्रसवपूर्व महीला की सेवा<br>Preparation for delivery C<br>प्रसव की तैयारी<br>Healthcare facility services D<br>सेहत सम्बन्धी सेवा<br>Jsy, jssk, or other scheme E<br>जननी सुरक्षा योजना/जननी षिषु सुरक्षा कार्यक्रम या अन्य योजना<br>Clean and safe deliveries F<br>साफ और सुरक्षित जन्म<br>Postpartum care of mothers G<br>प्रसव बाद महिला की सेवा<br>What to do when newborn babies have emergencies H<br>क्या करना<br>है जब नये जन्मे बच्चे को परेशानी हो<br>How to keep babies healthy I<br>बच्चे को स्वस्थ कैसे रखना है<br>Breastfeeding J<br>स्तनपान<br>Complementary feeding K<br>पूरक आहार<br>Immunizations L<br>टीकाकरण<br>Family planning M<br>परिवार नियोजन<br>Sanitation/hygiene N<br>स्वच्छता<br>other (specify) X<br>अन्य(स्पष्ट करें)		
**908A**	Are there any women aged 15-49 in your household who are not members of the community group ?<br>क्या आपके घर में 15-49 वर्ष के बीच कोई महिला है जो समूह की सदस्य नहीं है?	Yes हाँ______________1<br>No नहीं_______________2		
**908B**	How many such women are there ?<br>ऐसी कितनी महिलाएं आपके घर में हैं?	Number __________<br>संख्या		
**908C**	Have you ever told or discussed health related issues with this woman in your household who is not a member of the	Yes हाँ______________1<br>No_ नहीं______________2		

	community group ?<br><br>क्या आपने कभी आपके घर की उस महिला से जो समूह की सदस्य नही है उसे स्वास्थ्य सम्बंधित बातें बताई है?			

**909**	Did any of the front line health workers (eg., ASHA, ANM, AWWs) attend any of the meetings<br>क्या स्वास्थ्य कार्यकर्ता जैसे (आषा, एएनएम, आंगनवाडी कार्यकर्ता) ने कभी समूह चर्चा में भाग लिया?	Yes<br>No<br>I am not aware of their attendance<br>मुझे उनकी उपस्थिति के बारे में पता नहीं	1<br>2<br>3	► 911<br>► 911<br>यदि प्रश्न संख्या 909 में 2 या 3 कोड हुआ है तो प्रश्न संख्या 911 पर जाएँ
**910**	Who attended recently?<br>हाल के समय में इन स्वास्थ्य कर्ताओं में से किसने समूह चर्चा में भाग लिया?<br>एक से ज्यादा उत्तर संभव हैं	ASHA आषा<br>ANM एएनएम<br>AWW आंगनवाडी कार्यकर्ता<br>Other अन्य	A<br>B<br>C<br>X	
**910b**	Have any of the FLWs (ANM, ASHA, AWW) taught you about pregnancy or child care through « mobile kunji » ?<br><br>क्या किसी आशा, ए एन एम्, या आंगनवाडी वर्कर ने आपको प्रसव अथवा बच्चे की देखभाल के बारे में मोबाइल कुंजी के द्वारा समझाया है?	Yes हाँ<br>No नहीं	1<br>2	
**911**	Did you pay any membership fee for joining the group?<br>क्या आपको समूह में शामिल होने के लिए किसी सदस्यता शुल्क का भुगतान करना पड़ा?	Yes हां<br>No नहीं	1<br>2	
**912**	Do you contribute to savings scheme of the group?<br>क्या आप समूह की बचत योजना में योगदान करती हैं?	Yes हां<br>No नहीं<br>No such scheme समूह में पैसा जमा नहीं होता है	1<br>2<br>3	
**913**	Have you taken any loan from the community group?<br>क्या आपने समुदायिक समूह से कोई कर्ज लिया है?	Yes हां<br>No नहीं	1<br>2	► 916<br>यदि प्रश्न संख्या 913 में 2

				कोड हुआ है तो प्रश्न संख्या 916 पर जाएँ
**914**	How much loan did you take?<br>आपने समूह से कितना कर्ज लिया है?	Amount in rupees<br>राषि रूपये में	__________	
**915**	What is the monthly interest rate?<br>मासिक ब्याजदर कितना है?	Amount for 100 rupees loan<br>100 कर्ज के लिए राषि	_________	
**915a**	Did you repay (are repaying) the loan?<br>क्या आपने यह क़र्ज़ चूका दिया/चूका रहे है?	Yes हाँ<br>No नहीं	1<br>2	
**916**	Did you take any loan from an outside money lender?<br>क्या आपने महाजन या साहूकार से कर्ज लिया है?	Yes हां<br>No नहीं	1<br>2	► 919<br>यदि प्रश्न संख्या 916 में 2 कोड हुआ है तो प्रश्न संख्या 919 पर जाएँ
**917**	How much did you take?<br>आपने महाजन या साहूकार से कितना कर्ज लिया है?	Amount in rupees<br>राषि रूपये में	_________	
**918**	What is the monthly interest rate?<br>मासिक ब्याजदर कितना है?	Amount for 100 rupees loan<br>100 कर्ज के लिए राषि	_________	
**919**	Are you a member of any of the following groups?<br>क्या आप निम्नलिखित समूहों में से किसी के सदस्य हैं	 VHSC वी एच एस सी (ग्राम स्वास्थय और स्वछ्ता कमिटी)<br>Panchayat पंचायत<br>Rogi kalyan samiti रोगी कल्याण समिति<br>Reproductive health group प्रजनन स्वास्थ्य समूह<br>Other Self-help group स्वयं सहायता समूह	हाँ | नहीं<br>1 | 2<br>1 | 2<br>1 | 2<br>1 | 2<br>1 | 2	
**919f**	Does any member of this household have a bank account or post-office account?<br>क्या इस परिवार के किसी भी सदस्य के पास बैक में या पोस्ट ऑफिस में खाता है ?	Yes हाँ ....1<br>No नहीं ....0<br>Don’t know पता नहीं 98		

**919g**	Is any member of this household covered by a health scheme or health insurance?<br>क्या इस परिवार का कोई सदस्य किसी स्वास्थ्य योजना या स्वास्थ्य इंन्शयोरेन्स/बीमा से जुडा हुआ है?	Yes हाँ.......................................1<br>No नहीं ....0<br>Don't know पता नहीं 98	Skip to 920
**919h**	What type of health scheme or health insurance?<br>किस प्रकार की स्वास्थ्य योजना या स्वास्थ्य इंन्शयोरेन्स/बीमा से जुडे है ?<br><br>**PROBE:** Any others?<br>कोई अन्य<br><br>**CODE ALL THAT APPLY**<br>**जो लागू हो सभी को कोड करें**	Employees State Insurance........1<br>Scheme (ESIS).................................<br>अन्य इम्पलॉयी स्टेट इंन्थ्योरेन्स स्कीम्स<br>Central Government<br>Health Scheme (CGHS) 2.......................<br>इंन्थ्योरेन्स सैंट्रल गवर्नमेन्ट हेल्थ स्कीम्स<br>Community Health Insurance<br>Programme 3 ....<br>कम्यूनिटी हेल्थ इंन्थ्योरेन्स प्रोगाम<br>Other Health Insurance<br>THROUGH EMPLOYER 4......................<br>नियोक्ता द्वारा अन्य स्वास्थ्य बीमा<br>Medical Reimbursement<br>From Employer 5 ....<br>नियोक्ता द्वारा मैडिकल खर्चो की अदायगी<br>Other Privately Purchased<br>Commercial Health Insurance 6..............<br>अन्य प्राइवेट खरीदे गये स्वास्थ्य बीमा<br>Other (Specify)<br>..<br>8<br>अन्य (स्पष्ट करें)	

	**Leadership समूह के नेतृत्व से सम्बंधित**			
**920**	Does your group have a leader ('President')?<br>क्या अपके समूह में कोई लीडर है (प्रसीडेंट)	Yes हां<br>No नहीं	1<br>2	► 929<br>यदि प्रश्न संख्या 920 में 2 कोड हुआ है तो प्रश्न संख्या 929 पर जाएँ

**920A**	Who selected the leader?<br>लीडर किसने चुना?	Members of our own group अपने स्वयं समूह के सदस्य<br>Local NGO representative स्थानीय गैर सरकारी संगठन के प्रतिनिधि<br>Government representative सरकार के प्रतिनिधि<br>Other अन्य (स्पष्ट करें)<br>Do not know पता नहीं	1<br>2<br>3<br>8<br>9

**921**	Has the leadership of your group	Yes हां	1	

	`changed anytime till today?<br>क्या नेतृत्व में आज तक कभी बदलाव हुआ?	No नहीं			2	
	**How do you rate the leader on the following issues:**<br>**निम्नलिखित मुद्दों पर आप अपने लीडर का कैसे मुल्यांकन करेंगे?**	Very poor<br>बहुत खराब	Somewhat<br>थोडा बहुत	Good<br>अच्छा	Very Good<br>बहुत अच्छा	
**922**	Organising regular meetings<br>नियमित बैठकों का आयोजन	1	2	3	4	
**923**	Ensuring regular attendance of the members in meetings<br>बैठकों में सदस्यों कि नियमित उपस्थिति सुनिष्चित करना	1	2	3	4	
**924**	Addressing the needs/requests of the community group members<br>सामुदायिक सदस्यों के जरूरतों/अनुरोधों को पूरा करने का प्रयास करना	1	2	3	4	
**925**	Encouraging group members to talk about their concerns openly<br>समूह के सदस्यों को उनकी **जरूरतों/परेशानियों** के बारे में **बात करने के लिए** प्रोत्साहित करना	1	2	3	4	
**926**	Presenting community groups' concerns to relevant external stakeholders<br>सामुदायिक समूह के **मुद्दों** को बाहरी हितधारकों जैसे कि पंचायत, स्वास्थ्य के अधिकारियों, या किसी अन्य सरकारी/गैर सरकारी संस्था के अधिकारियों के सामने पेश करना	1	2	3	4	
**927**	Sharing of information she receives from external resources at the meetings.<br>बाहरी संसाधनों से मिली जानकारी को **समूह के सदस्यों** को बताना	1	2	3	4	
**928**	Resolving conflicts between members of the community group<br>समूदायिक समूह के सदस्यों की आपसी मतभेद/कलह को हल करना	1	2	3	4	

**<u>Sense of ownership</u>** <u>समूह के प्रति अपनी जिम्मेदारी से सम्बंधित</u>					
**934**	How responsible do you feel for achieving the objectives of your group?<br>अपने समूह के उद्देष्यों को प्राप्त करने के लिए आप अपने आप को कितना जिम्मेदार महसूस करती हैं?	Not at all बिल्कूल भी नहीं<br>Somewhat कुछ हद तक<br>Very much बहुत हद तक<br>Completely पूरी तरह से	1<br>2<br>3<br>4		

**<u>Social Cohesion:</u> Sense of belonging समूह में सामाजिक सामंजस्य और अपनेपन की भावना**			
**How much do you agree or disagree with the following statements: निम्नलिखित वाक्यों से आप कितनी सहमत या असहमत हैं**			
**935**	I feel that I belong to this community group.<br>मुझे लगता है कि मैं इस सामूदायिक समूह से हूँ	Strongly agree पूरी तरह से सहमत<br>Agree सहमत<br>Disagree असहमत<br>Strongly disagree पूरी तरह से असहमत	1<br>2<br>3<br>4
**936**	I would rather go with a different community group.<br>मुझ लगता है कि मूझे दूसरे सामूदायिक समूह **से जुड़ना चाहिए**	Strongly agree पूरी तरह से सहमत<br>Agree सहमत<br>Disagree असहमत<br>Strongly disagree पूरी तरह से असहमत	1<br>2<br>3<br>4
**937**	I would rather prefer this community group than any others I know of.<br>मैं इसी सामूदायिक समूह को पसंद करूंगी अन्य समूहो के मुकाबले	Strongly agree पूरी तरह से सहमत<br>Agree सहमत<br>Disagree असहमत<br>Strongly disagree पूरी तरह से असहमत	1<br>2<br>3<br>4
**938**	Members in this community group are all striving for the same goals.<br>इस सामूदायिक समूह के सदस्य सब एक ही लक्ष्य के लिए प्रयास कर रहे हैं।	Strongly agree पूरी तरह से सहमत<br>Agree सहमत<br>Disagree असहमत<br>Strongly disagree पूरी तरह से असहमत	1<br>2<br>3<br>4

**939**	Everyone in our community group wants to pursue their own goals rather than working for the good of the community.<br>हमारे सामूदायिक समूह के सभी सदस्य समुदाय की भलाई के लिए काम करने के बजाए अपने लक्ष्यों को आगे लाने की कोशिश करते है।	Strongly agree पूरी तरह से सहमत<br>Agree सहमत<br>Disagree असहमत<br>Strongly disagree पूरी तरह से असहमत	1<br>2<br>3<br>4
**940**	I can count on fellow community group members if I need to borrow money<br>मैं अपने सामुदायिक समुह के साथीयों पर भरोसा कर **सकती** हूँ यदि मुझे पैसे उधार लेने की जरूरत पडी तो.	Strongly agree पूरी तरह से सहमत<br>Agree सहमत<br>Disagree असहमत<br>Strongly disagree पूरी तरह से असहमत	1<br>2<br>3<br>4
**941**	I can count on fellow community group members to accompany me to the doctor or hospital<br>मैं अपने सामुदायिक समुह के साथीयों पर भरोसा कर **सकती** हूँ वे मेरे साथ डाक्टर या अस्पताल आ सकते है	Strongly agree पूरी तरह से सहमत<br>Agree सहमत<br>Disagree असहमत<br>Strongly disagree पूरी तरह से असहमत	1<br>2<br>3<br>4
**942**	I can count on fellow community group members to talk about my problems<br>मैं अपने सामुदायिक समुह के साथीयों पर भरोसा कर **सकती** हूँ और मैं उनको मेरी समस्याओं के बारे में बता **सकती** हूँ	Strongly agree पूरी तरह से सहमत<br>Agree सहमत<br>Disagree असहमत<br>Strongly disagree पूरी तरह से असहमत	1<br>2<br>3<br>4
**943**	Community group members worry about themselves only<br>सामुदायिक समूह के सदस्यों को **सिर्फ** खुद के बारे में चिंता है	Strongly agree पूरी तरह से सहमत<br>Agree सहमत<br>Disagree असहमत<br>Strongly disagree पूरी तरह से असहमत	1<br>2<br>3<br>4
**944**	I can count on fellow community group members if I need advice<br>मैं अपने सामुदायिक समुह के साथीयों पर भरोसा कर सकती हूँ यदि मुझे किसी सलाह की जरूरत है	Strongly agree पूरी तरह से सहमत<br>Agree सहमत<br>Disagree असहमत<br>Strongly disagree पूरी तरह से असहमत	1<br>2<br>3<br>4
**<u>Social Cohesion:</u> Network cohesion सामाजिक एकजुटता/सामंजस्यः नेटवर्क सामंजस्य**			
**951**	With how many different members of your community		

	group did you talk separately about the issue that was discussed in the last meeting?<br>आपने अपने सामुदायिक समूह के कुल कितने सदस्यों से पिछली बैठक में चर्चा किए हुए मुद्दों पर अलग अलग बातचीत **की** ?	Number<br>संख्या	________<br>_
**952**	When you have a problem or worry, how often do you let someone else in the community group know about it?<br>जब आपको किसी प्रकार की समस्या या चिंता होती है तो कितनी बार आप **सामुदायिक समूह की** किसी और **सदस्य** को इस बारे में बताते है	Never कभी नहीं<br>Rarely शायद ही कभी<br>Sometimes कभी कभी<br>Usually आमतौर पर<br>Always हमेशा	1<br>2<br>3<br>4<br>5
**953**	How often can you rely on someone in your community group for help when you have a serious problem?<br>कितनी बार आप मदद के लिए **अपने समूह की सदस्यों** पर भरोसा कर सकते है जब आप गंभीर समस्या से परेषान है?	Never कभी नहीं<br>Rarely शायद ही कभी<br>Sometimes कभी कभी<br>Usually आमतौर पर<br>Always हमेशा	1<br>2<br>3<br>4<br>5

	**Enabling environment**			
**954**	Do you have equal access to a health facility like everyone else (for e.g., the women from forward castes)?<br>आपके हिसाब से क्या आप स्वास्थ्य सुविधाओं का प्रयोग बाकी की महिलाओं के जैसे ही कर सकती हैं (उदाहरण के लिए सामान्य या ऊँची जाति की महिलाएं)?	Yes हां<br>No नहीं	1<br>2	

955	Would you say the following people treat you fairly like they do other people (for e.g., women from forward castes)?<br>क्या आप कहेंगी कि निम्नलिखित लोग आपसे वैसा ही व्यवहार करते हैं जैसा अन्य लोगों के साथ करते हैं (उदाहरण के लिए सामान्य या ऊँची जाति की महिलाएं)?	Not at all fairly<br>बिलकुल नहीं	Somewhat fairly<br>कुछ हद तक	Very fairly<br>काफी हद तक	Completely fairly<br>बिलकुल वैसा ही व्यवहार	NA<br>लागू नहीं
A	Doctors in government hospital<br>सरकारी अस्पताल में डाक्टर	1	2	3	4	9
B	ASHAs in your village<br>अपने गांव में आशा	1	2	3	4	9
C	AWWs at the anganwadi centers<br>आंगनवाडी कार्यकर्ता आंगनवाडी केन्द्र में	1	2	3	4	9
D	ANM<br>एएनएम	1	2	3	4	9
E	Nurses in government hospital<br>सरकारी अस्पताल में नर्स	1	2	3	4	9
F	Doctors in private hospital<br>निजी अस्पताल में डाक्टर	1	2	3	4	9
G	Members of a panchayat<br>पंचायत के सदस्य	1	2	3	4	9
H	Sarpanch<br>स्रपंच	1	2	3	4	9

956	**I'd now like to ask you about the ASHA. Would you say the ASHA:**<br>**अब मैं आपसे आशा के बारे में पूछना चाहूंगी | क्या आप कहेंगी कि आशाः**			
A	Treats you with respect.<br>आपके साथ आदरपूर्वक व्यवहार करती है	Yes हां<br>No नहीं	1<br>2	
B	Directs you to appropriate health service providers<br>आपको उचित स्वास्थ्य सेवा प्रतादाओं के पास भेजती है	Yes हां<br>No नहीं	1<br>2	
C	Responds quickly to emergency situations<br>आपात स्थिति में तुरंत उपलब्ध होती है	Yes हां<br>No नहीं	1<br>2	
D	Is available when you need her<br>उपलब्ध रहती है जब आपको जरूरत होती है	Yes हां<br>No नहीं	1<br>2	
957	**I'd now like to ask you about the AWW. Would you say the AWW**<br>**अब मैं आपसे आंगनवाडी कार्यकर्ता के बारे में पूछना चाहूंगी | क्या आप कहेंगी कि आंगनवाडी कार्यकर्ता :**			
A	Treats you with respect.<br>आपके साथ आदरपूर्वक व्यवहार करती है	Yes हां<br>No नहीं	1<br>2	
B	Directs you to appropriate health service providers<br>आपको उचित स्वास्थ्य सेवा प्रदाओं के पास भेजती है	Yes हां<br>No नहीं	1<br>2	
C	Responds quickly to emergency situations<br>आपात स्थिति में तुरंत उपलब्ध होती है	Yes हां<br>No नहीं	1<br>2	
D	Is available when you need her<br>उपलब्ध रहती है जब आपको जरूरत होती है	Yes हां<br>No नहीं	1<br>2	

958	**How much do you think the activities of your community group have contributed to a positive change in:**<br>**आपके हिसाब से, सामूदायिक समूह द्वारा किए गए गतिविधियों का निम्नलिखित के स्वास्थय सुविधाओं के इस्तेमाल, परिवार नियोजन, और साफ़-सफाई से सम्बंधित व्यवहार के अच्छे बदलाव में कितना योगदान है :**	Not at all बिलकुल नहीं	Somewhat थोडा बहुत	Very much काफी हद तक	Completely बहुत ज्यादा	
**A**	You आप में	1	2	3	4	
**B**	Members of your group समूह के सदस्यों में	1	2	3	4	
**C**	Members of other groups in your village गाँव के दूसरे समूहों के सदस्यों में	1	2	3	4	
**D**	People who are not members<br>उन लोगों में जो समूह के सदस्य नहीं हैं	1	2	3	4	

<u>Collective Efficacy सामूहिक प्रभावकरिता</u>

**959**	If there were problems that affected all or some of the community members, how many women would work together to deal with the problem:<br>यदि गॉव में ऐसी कोई समस्याएं हो जिसका असर समूह के कुल या कुछ सदस्यों पर पड सकता हो...**तो आपके अपने समूह से कुल** कितनी महिलाएं एक साथ मिलकर उस समस्या को सुलझाने के लिए काम करेंगी			
**A**	From your own community group<br>आपके अपने समूह से	No one कोई नहीं<br>Some कुछ<br>Most अधिकांष<br>All सब	1<br>2<br>3<br>4	
**B**	Women in the village but not from community groups<br>गॉव की अन्य महिलाएं जो समूह की सदस्य नहीं हैं	No one कोई नहीं<br>Some कुछ<br>Most अधिकांष<br>All सब	1<br>2<br>3<br>4	

**960**	**How confident are you that the members of your community can work together to achieve the following goals:**<br>**आपको कितना विश्वास है कि आपके समुदाय के सदस्य निम्नलिखित लक्ष्य को पाने के लिए आपस में मिलजुलकर काम करेंगे:**		
**A**	Speaking up against the existing norms imposed by elderly or other groups on issues around mother and child health<br>मां और बच्चे के स्वास्थ्य **से सबंधित मुद्दों** पर **बुजुर्गो या अन्य लोगों द्वारा बनाये** गए नियमों के खिलाफ **आवाज उठाना**	Not at all confident बिल्कूल विश्वास नहीं<br>Somewhat confident थोड़ा विष्वास है<br>Very confident बहुत विष्वास है<br>Completely confident पूरी तरह से विष्वास है	1<br>2<br>3<br>4
**B**	Demanding services from healthcare facilities when they refuse support<br>स्वास्थ्य सुविधाओं से सेवाओं की मांग करना, जब वे आपको सुविधाएॅ नहीं दे रहे हों	Not at all confident बिल्कूल विश्वास नहीं<br>Somewhat confident थोड़ा विष्वास है<br>Very confident बहुत विष्वास है<br>Completely confident पूरी तरह से विष्वास है	1<br>2<br>3<br>4

**C**	Claiming rights/schemes from the government<br>सरकार से अपने अधिकार और अपने लिए योजनाओं की मांग करना	Not at all confident बिल्कूल विश्वास नहीं<br>Somewhat confident थोड़ा विष्वास है<br>Very confident बहुत विष्वास है<br>Completely confident पूरी तरह से विष्वास है	1<br>2<br>3<br>4
**D**	Increase safe practices around mother and child health (for eg., ensuring the delivery at hospital, immunization of children, seeking antenatal care)<br>मॉ और बच्चे के स्वास्थ्य से संबंधित सुरक्षित व्यवहारों को बढ़ावा देना (उदाहरण के लिए बच्चो का टीकाकरण, अस्पताल में प्रसव सुनिष्चित करना, प्रसव पूर्व देखभाल)	Not at all confident बिल्कूल विश्वास नहीं<br>Somewhat confident थोड़ा विष्वास है<br>Very confident बहुत विष्वास है<br>Completely confident पूरी तरह से विष्वास है	1<br>2<br>3<br>4

**<u>Collective agency</u>**			
**961**	**In the past 6 months, have you negotiated with the following in order to help a fellow community member:**<br>**पिछले 6 महीनो में, समुदाय के सदस्यों के मदद के लिए आपने निम्नलिखित में से किसी से बातचीत किया या मदद के लिए उठ खड़ी हुई**		
**A**	Healthcare center staff e.g. doctor, nurse<br>स्वास्थ्य केन्द्र के कर्मचारी जैसे की डाक्टर, नर्स	Yes हां<br>No नहीं<br>NA लागू नहीं	1<br>2<br>3
**B**	Frontline workers e.g. ASHA, AWW, ANM<br>स्वास्थय कार्यकर्ताओं जैसे कि आषा, आंगनवाडी कर्मचारी, ए एन एम	Yes हां<br>No नहीं<br>NA लागू नहीं	1<br>2<br>3
**C**	Local administration (e.g. police, civil supplies, etc.)<br>स्थानीय प्रषासन (जैसे कि पुलिस, आम लोगों की आवष्यकताओं की चीज का वितरण करने वाले संस्थान इत्यादि)	Yes हां<br>No नहीं<br>NA लागू नहीं	1<br>2<br>3
**962**	In the past 6 months, have you participated in any of the community groups' protests or demands that concerned the women like you?<br><br>पिछले 6 महीनो में, क्या आपने सामूदायिक समूह द्वारा किये गए किसी ऐसे कार्य में भाग लिया जिसमे आप जैसी महिलाओं से सम्बंधित मुद्दों पर किसी का विरोध किया गया था या कुछ मांग की गयी थी	Yes हां<br>No नहीं<br>NA लागू नहीं	1<br>2<br>3
**<u>Collective action</u>**			
**963**	**Has your group come together in the past 6 months to demand the following:**<br>**निम्नलिखित मांग के लिए क्या आपके समूह की महिलाएं एकजुट होती हैं?:**		
**A**	Better health services for mother and child from local health center<br>स्थानीय स्वास्थ्य केन्द्र से मा और बच्चे के लिए बेहतर स्वास्थ्य सेवा	Yes हां<br>No नहीं	1<br>2
**B**	Services/schemes meant for poor e.g. for the JSY scheme<br>गरीबो और जरूरतमंदों के लिए बनी योजनाओं के लिए (जैसे कि आंगनवाडी केंद्र से मिलाने वाली पोषाहार योजना)	Yes हां<br>No नहीं	1<br>2

**C**	On-time delivery of incentives from the government<br>सरकार की ओर से प्रोत्साहन राषि का समय पर भुगतान (जैसे कि जननी एंव बाल सुरक्षा योजना)	Yes हां<br>No नहीं	1<br>2
**D**	Opening of bank accounts<br>बैंक खातो को खोलना	Yes हां<br>No नहीं	1<br>2
**E**	Availability of family planning services<br>परिवार नियोजन सेवाओं की उपलब्धता	Yes हां<br>No नहीं	1<br>2
**F**	Supply of safe drinking water<br>सुरक्षित पीने के पानी की आपूर्ति	Yes हां<br>No नहीं	1<br>2
**G**	Supply of sufficient water for household purposes<br>घरेलू कामों के लिए पर्याप्त पानी की आपूर्ति	Yes हां<br>No नहीं	1<br>2
**H**	Supply of sanitation services e.g. latrine facilities<br>स्वच्छता सेवाओं की आपूर्ति उदाहरण के लिए शौचालय की सुविधा	Yes हां<br>No नहीं	1<br>2
**964**	The last time you had some problem during pregnancy or child birth, did the other women from your group help you?<br>पिछली बार गर्भावस्था या बच्चे के जन्म के दौरान हुई किसी समस्या में क्या आपको समुह के महिलाओं से कोई मदद मिली थी?	Yes हां<br>No नहीं<br>Never had a problem कोई समस्या नहीं हुई थी<br>Not Applicable लागू नहीं	1<br>2<br>3<br>9
**965**	Did the other women from your group help you, when you needed specific services for your or your child's health?<br>जब आपको अपने और बच्चे के स्वास्थ्य के लिए किसी प्रकार की मदद की जरूरत थी तो क्या आपके समुह के महिलाओं ने आपकी मदद **की थी?**	Yes हां<br>No नहीं<br>Never had a problem कोई समस्या नहीं हुई थी<br>Not Applicable लागू नहीं	1<br>2<br>3<br>9
**966**	Did any women from the village/panchayat (but not from your community) help you, when you needed specific services during pregnancy or child birth?<br>क्या आपके गांव की किसी महिला जो आपके समूह की सदस्य नहीं थी, ने गर्भावस्था या बच्चे के जन्म के दौरान आपकी मदद **की थी?**	Yes हां<br>No नहीं<br>Never had a problem कोई समस्या नहीं हुई थी<br>Not Applicable लागू नहीं	1<br>2<br>3<br>9

**SELF EFFICACY AND SELF CONFIDENCE स्व प्रभावकरिता और आत्मविश्वास**				
**967**	How confident do you feel speaking your opinion in the community group?<br>आप कितने विष्वास से अपनी राय अपने सामूदायिक समूह के सामने रखती हैं?	Not at all confident बिल्कूल विश्वास नहीं<br>Somewhat confident थोड़ा विष्वास है<br>Very confident बहुत विष्वास है<br>Completely confident पूरी तरह से विष्वास है	1<br>2<br>3<br>4	
**968**	How confident are you in recognizing the danger signs of pregnancy, delivery?<br>आप कितने विष्वास से गर्भावस्था और प्रसव के खतरे के संकेत को पहचानती हैं?	Not at all confident बिल्कूल विश्वास नहीं<br>Somewhat confident थोड़ा विष्वास है<br>Very confident बहुत विष्वास है<br>Completely confident पूरी तरह से विष्वास है	1<br>2<br>3<br>4	
**969**	How confident are you that you can go to the government health centers to get reproductive health services?<br>आपको कितना विष्वास है कि आप सरकारी स्वास्थ्य केन्द्र जाकर स्वास्थ्य सेवाओं (मॉ और बच्चों के स्वास्थ्य से संबंधित, परिवार नियोजन से संबंधित सुविधाऍं) का लाभ ले सकती है	Not at all confident बिल्कूल विश्वास नहीं<br>Somewhat confident थोड़ा विष्वास है<br>Very confident बहुत विष्वास है<br>Completely confident पूरी तरह से विष्वास है	1<br>2<br>3<br>4	
**970**	How confident do you feel in giving advice to women from your own community on the steps	Not at all confident बिल्कूल विश्वास नहीं<br>Somewhat confident थोड़ा विष्वास है	1<br>2	

	towards safe practices during and after pregnancy?<br>आप कितने विश्वास के साथ अपने समुदाय की महिलाओं को गर्भावस्था के दौरान और गर्भावस्था के बाद की देखभाल के बारे में सलाह दे सकती है?	Very confident बहुत विष्वास है<br>Completely confident पूरी तरह से विष्वास है	3<br>4	
**971**	How confident do you feel in giving advice to women in the village (who do not belong to your caste on the steps towards safe practices during and after pregnancy?<br>आप कितने विष्वास के साथ ऐसी महिलाओं को जो आपके समुदाय की सदस्य नहीं है, गर्भावस्था के दौरान और गर्भावस्था बाद की देखभाल के बारे में सलाह देती है?	Not at all confident बिल्कूल विश्वास नहीं<br>Somewhat confident थोड़ा विष्वास है<br>Very confident बहुत विष्वास है<br>Completely confident पूरी तरह से विष्वास है	1<br>2<br>3<br>4	

**INDIVIDUAL AGENCY/CONTROL**				
**972**	Do you need permission (from your husband or in-laws) for each of the following:<br>क्या आपको निम्नलिखित कार्यो के लिए अनुमति लेनी पडती है:		Yes	No
		Go to a movie<br>फिल्म देखने जाने के लिए	1	2
		Go to the doctor<br>डाक्टर के पास जाने के लिए	1	2
		Buy new clothes for yourself<br>अपने लिए नया कपडा खरीदने के लिए	1	2
		Travel to the town<br>शहर जाने के लिए	1	2
		To participate in group meetings<br>समूह की बैठकों में भाग लेने के लिए	1	2
		To take child to health care center<br>बच्चे को स्वास्थय केंद्र ले जाने के लिए	1	2
		To use family planning methods /contraception<br>परिवार नियोजन की विधियों/गर्भनिरोध के उपयोग के लिए	1	2

**MENTAL HEALTH STATUS**			
**973**	Do you make an effort to take care of your health (for example, do you eat well, sleep well, go to the doctor when sick, etc.)<br>क्या आप अपनी सेहत का ख्याल रखने के लिए प्रयास करते है (उदाहरण . के लिए: अच्छी तरह से खाना, अच्छी तरह से सोना, जब आप बिमार होते है तब डाक्टर के पास जाना, आदि)	Never कभी नहीं<br>Rarely शायद ही कभी<br>Sometimes कभी कभी<br>Usually आमतौर पर<br>Always हमेषा	1<br>2<br>3<br>4<br>5

**974**	Over the last 2 weeks, how often have you been bothered by any of the following problems:<br>पिछले 2 सप्ताह में, कितनी बार आप निम्न समस्याओं मे से एक बार भी परेषान हुई?	Not at all<br>कभी परेशानी नहीं हुई	Several days<br>कई दिन	More than half days<br>आधा दिन से अधिक	Nearly every day<br>लगभग हर दिन	
**A**	Little or no interest or pleasure in doing things<br>कार्य करने में थोड़ी रूचि या कार्य करने में बिल्कुल खुषी नहीं	1	2	3	4	
**B**	Feeling down, depressed, or hopeless उदास या निराष महसूस करना	1	2	3	4	

FOOD SECURITY

Now, I will ask some questions about food available to eat in your household.<br>अब मै इस परिवार के लिए उपलब्ध खाने के लिए भोजन के बारे मे कुछ सवाल पूछुँगी		
1001	In the past four weeks, did your household ever run out of money to buy food?<br>पिछले चार हफ्तों में क्या आपके परिवार में कभी भोजन खरीदने के लिए पैसे की कमी हुई है?	Yes हाँ...1<br>No नहीं 0
1002	In the past four weeks, did you ever limit the types of food you fed your children because you did not have enough money to buy food for a meal?<br>पिछले चार सप्ताह में क्या आपने भोजन के प्रकार कम कीए है जो आप अपने बच्चों को खिलाती हैं क्योंकि आपके पास भोजन या खाना खरीदने के लिए पैसे नहीं थे?	Yes हाँ...1<br>No नहीं 0
1003	In the past four weeks, did you cut the size of meals or skip meals because there was not enough money for food?<br>पिछले चार सप्ताह में क्या आपने भोजन के लिए पर्याप्त पैसा न होने के कारण कभी अपने आहार की मात्रा को कम किया हैं या फिर आहार नहीं लिया है?	Yes हाँ ....1<br>No नहीं 0
1004	In the last four weeks, did you or any household member go to sleep at night hungry because there was not enough food?<br>क्या पिछले 4 सप्ताह मे आप या आपके परिवार का कोई सदस्य खाना पर्याप्त नही होने के कारण से रात मे भूखा सोया था	Yes हाँ...1<br>No नहीं 0

MARITAL VIOLENCE

Now I would like to ask you questions about some other important aspects of a woman's life. I know that some of these questions are very personal. However, your answers are crucial for helping to understand the condition of women in India. Let me assure you that your answers are completely confidential and will not be told to anyone and no one else will know that you were asked these questions.<br>अब मैं आपसे महिलाओं के जीवन से संबंधित कुछ महत्वपुर्ण पहलुओं के बारे में कुछ प्रश्न पुछुंगी। मैं जानती हुं कि इनमें से कुछ प्रश्न बहुत व्यक्तिगत हैं। आपके उत्तर हमें भारतीय महिलाओं की स्थिति को पहचानने के लिए काफी महत्वपुर्ण हैं। मैं आपको यह विश्वास दिलाना चाहती हुं कि आपके उत्तर पुरी तरह गोपनिय हैं और वह किसी को भी नही बताये जायेंगे और किसी को यह भी पता नही चलेगा कि आपसे यह प्रश्न पुछे गये थे?					
1101	First, I am going to ask you about some situations which happen to some women. Please tell me if these apply to your relationship with your (last) husband.<br>पहले मैं आपसे कुछ स्थितियों के बारे में पूछूंगा जो महिलाओं के साथ घटती हैं। मुझे बतायें कि क्या यह आपके पति (पिछले) के संबंधों के मामले में भी ऐसा ही होता है?		Yes<br>ळां	No<br>न्हीं	Dk<br>मालूम नहीं
	a. He (does/did) not permit you to meet your female friends.<br>ए. वह आपको आपकी सहेलियों से नहीं मिलने देता/मिलने देता था।	Not.meet.friends<br>मित्रों से मिलने पर रोक	1	2	98
	b. He (tries/tried) to limit your contact with your family.<br>बी. वह आपके परिवार के साथ आपके संपर्क पर रोक लगाने की कोशिश करता है/करता था।	No family<br>परिवार से संपर्क नहीं	1	2	98
	c. He (does/did) not trust you with any money.<br>सी. पैसे को लेकर वह आप पर भरोसा नहीं करता/करता था	Money<br>पैसा	1	2	98
	d. He (insists/insisted) on knowing where you (are/were) at all times.<br>डी वह यह जानने पर (जोर देता है/देता था) कि आप हर समय कहां रहीं/कहां थीं?	Where you are<br>आप कहां थीं	1	2	98
	e. He frequently (accuses/accused) you of being unfaithful.<br>ई. वह अक्सर आप पर बेवफा होने का आरोप लगाता है/लगाता था?	Accuses<br>आरोप लगाना	1	2	98
1102	Does (did) your husband drink alcohol?<br>**क्या आपका पति शराब पीता हैं (था)?**	YES हां ....1<br>NO…नहीं ....0 ► 1104<br>**यदि प्रश्न संख्या 1102 में 2 कोड हुआ है तो प्रश्न संख्या 1104 पर जाएँ**			
1103	How often does (did) he get drunk: often, only sometimes, or never?<br>**वह कितनी अंतराल पर शराब पीता हैं। अक्सर, कभी कभी या कभी नहीं**	Often **अकसर**...................1<br>Sometimes **कीाी कीाी**...........2<br>Never **कभी नही**............3			
1104	Now if you will permit me, I need to ask some more questions about your relationship	Yes<br>हां	No<br>न्हीं	**Ask if yes in 1104 यह प्रश्न तभी पूछें**	

	with your (last) husband.<br>(Does/did) your (last) husband ever:<br>अब अगर आप मुझे इजाजत दें तो मैं आपसे आपके पति (पिछले पति) के साथ आपके संबंधों के बारे में कुछ सवाल पूछना चाहूंगी।<br><br>क्या आपका वर्तमान पति (पिछला पति) निम्नलिखित कार्य करता है/था?			**यदि 1104 में उत्तर "हाँ" हो**<br>1104a.How often did this happen during the last 12 months: often, only sometimes, or not at all?<br>पिछले 12 महीनों में ऐसा कब–कब हुआ; अक्सर, कभी–कभी, कभी नहीं?<br>Often...................1<br>Sometimes...........2<br>Not at all............3<br>अक्सर.........................1<br>कभी–कभी..................2<br>कभी नहीं...................3
	a. Say or do something to humiliate you in front of others?<br>ए. दूसरों के सामने आपको अपमानित करने के लिए कुछ कहता या करता था?	1	2	|___|
	b. Threaten to hurt or harm you or someone close to you?<br>बी. आपको या आपके किसी निकट के व्यक्ति को चोट या नुकसान पहुंचाने की धमकी देता था?	1	2	|___|
	c. Insult you or make you feel bad about yourself?<br>सी. आपकी बेइज्जती करता था या आपको बुरा महसूस करवाता था?	1	2	|___|
1105	(Does/did) your (last) husband ever do any of the following things to you:<br>क्या आपके वर्तमान या पिछले पति ने आपके निम्नलिखित में कुछ किया/किया था?	Yes	No	**Ask if yes in 1105 यह प्रश्न तभी पूछें यदि 1105 में उत्तर "हाँ" हो**<br><br>1105a.How often did this happen during the last 12 months: often, only sometimes, or not at all?<br>वी3ए. पिछले 12 महीनों में ऐसा कब–कब हुआ; अक्सर, कभी–कभी, कभी नहीं?<br>Often...................1<br>Sometimes...........2<br>Not at all............3<br>अक्सर.........................1<br>कभी–कभी..................2<br>कभी नहीं...................3
	a.Slap you?<br>ए. आपको थप्पड़ मारा?	1	2	|___|
	b. Twist your arm or pull your hair?<br>बी. आपकी बांह मरोड़ी, बाल खींचे?	1	2	|___|
	c. Push you with his fist, shake you, or throw something at you?<br>सी. आपको हाथ से धक्का दिया, झकझोरा या आप पर कुछ फेंक कर मारा?	1	2	|___|
	d. Kick you, drag you or beat you up?<br>ई. आपको लात मारी, खींचा या मारापीटा	1	2	|___|

	e. Try to choke you or burn you on purpose?<br>एफ. आपका गला दबाने, आपको जलाने की कोशिश की?	1	2	|___|
	f. Physically force you to have sexual intercourse with him even when you did not want to?<br>एच. आपके न चाहने पर भी आपके साथ जबर्दस्ती यौन संपर्क किया?	1	2	|___|
1106	**Ask if yes in 1105, else go to 1201**<br>Have you ever tried to seek help to stop your husband from doing this to you again?<br>क्या आपने पति को दोबारा ऐसे करने से रोकने के लिए सहायता प्राप्त करने की कोशिश की हैं?	Yes……………………………………………1<br>हां<br>Noनहीं…………………………………………2 ► 1201<br>**यदि प्रश्न संख्या 1106 में 2 कोड हुआ है तो प्रश्न संख्या 1201 पर जाएँ**		
1107	From whom have you sought help to stop this?<br>Anyone else?<br><br>आपने यह रोकने के लिए किससे सहायता मांगी?<br>कोई अन्य?<br><br>**RECORD ALL MENTIONED.**<br>**जो भी उल्लेख हो उसे दर्ज करें**	Own family..A<br>अपना परिवार<br>Husband's family..B<br>पति का परिवार<br>Current/last husband......................................C<br>वर्तमान / पिछला पति<br>Current/former boyfriend.............................D<br>वर्तमान / पिछला ब्वायफ्रेंड<br>Friend..E<br>दोस्त<br>Neighbour...F<br>पड़ौसी<br>Religious leader...G<br>धार्मिक नेता<br>Doctor/medical personnel...............................H<br>डाक्टर / चिकित्साकर्मी<br>Police..I<br>पुलिस<br>Lawyer...J<br>वकील<br>Social service organization....................K<br>सामाजिक सेवा संगठन<br>Other (specify)..X<br>अन्य (उल्लेख करें)		
1108	Did you report to someone about the violence that you have experienced?<br>**आप पर जो हिंसा हुई, क्या आपने उसकी कभी रिपोर्ट की?**	Yes हा<br>No नहीं		
1109	Have you ever hit, slapped, kicked or done anything else to physically hurt your husband at times when he was not already beating or physically hurting you?	Yes हां<br>No नहीं ► 1201<br>**यदि प्रश्न संख्या 1109 में 2 कोड हुआ है तो प्रश्न संख्या 1201 पर जाएँ**		

	क्या आपने कभी अपने पति को मारा, चाटा मारा, लात मारी अथवा किसी और तरह से शारीरिक नुक्सान पहुचाया जबकि वह आपको मार नहीं रहे थे या कोई शारीरिक नुक्सान नहीं पंहुचा रहे थे?	
1110	In the last six months, how often have you done this to your husband?<br>**पिछले 6 माह में आपने कितनी बार अपने पति को मारा?**<br>***1= often* अक्सर *; 2= sometimes* कभी कभी*;*<br>*3=not at all* कभी भी नहीं*;***	1 2 3

SOCIAL ENTITLEMENTS
I will now ask you some questions related to government schemes. Please answer these questions as best as you can.

1201	Does this household have a BPL card/coupon?<br>क्या इस परिवार के पास बी पी एल कार्ड (लाल कार्ड)/कूपन है<br>**DESCRIBE BPL FOR CLARITY**.<br>**स्पष्टता के लिए बी पी एल के बारे में जानकारी दें।**	Yes हाँ...<br>No नहीं...<br>Don't Know पता नहीं 98			
1202	Does this household have a RSBY card? (also called Smart Card or Health Insurance Card)<br>क्या इस परिवार के पास आर. एस .बी. वाय. कार्ड (स्मार्ट कार्ड अथवा हेल्थ इन्सुरांस या स्वास्थय बीमा कार्ड) हैं?<br><br>DESCRIBE RSBY FOR CLARITY<br>**स्पष्टता के लिए आर. एस .बी. वाय के बारे में जानकारी दें।**	Yes हाँ...<br>No नहीं...<br>Don't Know पता नहीं98			
1203	Does this household have a NREGA card?<br>क्या इस परिवार के पास नरेगा का जॉब कार्ड है<br>DESCRIBE NREGA FOR CLARITY<br>**स्पष्टता के लिए नरेगा के बारे में**	Yes हाँ 1.....................................<br>No नहीं 2.....................................<br>Don't Know पता नहीं 98			

	जानकारी दें।				
	Have you or your family members availed of the following government schemes आपने अथवा आपके परिवार ने निम्न सरकारी योजना का लाभ उठाया है?	Yes हाँ	No नहीं	Aware but not availed it पता है पर इस्तेमाल नहीं किया	Not aware of the scheme योजना के बारे में पता नहीं
1204	Mukhyamantri Balika Poshak Yojana मुख्यमंत्री बालिका पोषक योजना	1	2	3	4
1205	Mukhyamantri Balika Cycle Yojana मुख्यमंत्री बालिका साइकिल योजना	1	2	3	4
1206	Mukhyamantri Kanya Suraksha Yojana मुख्यमंत्री कन्या सुरक्षा योजना	1	2	3	4
1207	Mukhyamantri Kanya Vivah Yojna मुख्यमंत्री कन्या विवाह योजना	1	2	3	4
1208	Mamta Scheme ममता योजना	1	2	3	4
1209	Sabla scheme (for girls 10-18 years old) सबला योजना (10-18 साल की लड़कियों के लिए)	1	2	3	4
1210	Saksham Scheme (for boys 10-18 years old) सक्षम योजना (10-18 साल के लडको के लिए)	1	2	3	4
1211	Indira Awas Yojana इंदिरा आवास योजना	1	2	3	4
1212	Aam Aadmi bima yojana आम आदमी बीमा योजना	1	2	3	4
1213	Indira Gandhi Matritiva Sahyog Yojana इंदिरा गांधी मत्रितिव योजना	1	2	3	4
1214	Anganwadi/Supplementay Nutrition Support (ICDS) अनागंवादी/परिषित पोषक सहायता	1	2	3	4
1215	Pradhan mantri Aadarsh Gram Yojana	1	2	3	4

	प्रधान मंत्री आदर्श ग्राम योजना				
1216	Sampoorna Grameen Rozgar Yojana संपूर्ण ग्रामीण रोज़गार योजना	1	2	3	4
1217	Swabhiman स्वाभिमान योजना	1	2	3	4

I will now ask you some questions about the saheli associated with your community group
अब आपसे आपके समूह (ग्रुप) की सहेली के बारे में कुछ सवाल पूछेंगें

	Did you receive advise from your saheli about the following<br>आपको सहेली ने कभी निम्न के बारे में सलाह दी है:	Yes हाँ	No नहीं
1301a	Early (within 3 months) registration of pregnancy<br>प्रसव के लिए शीघ्र (3 महीने के अन्दर)पंजीकरण		
1301b	Importance of at least 3 ANC visits<br>प्रसव के पहले देखभाल के लिए तीन बार चेकुप का महत्व		
1301c	Importance of consuming 90 IFA tablets during pregnancy<br>गर्भावस्था में ९० (90) आई ऍफ़ ए (IFA) गोलियां लेने का महत्व		
1301d	Importance of delivering your baby in a health facility<br>अस्पताल में प्रसव का महत्व		
1301e	Complete immunization of child<br>बच्चे को सभी टीके लगवाने का महत्व		
1301f	Giving ORS (oral rehydration solvent) to your child during diarrhoea.<br>डायरिया के समय बच्चे को ओ. र स. देने का महत्व		
1301g	Family planning<br>परिवार नियोजन		

1302	Last year we asked some women from your group some questions related to their pregnancy, other health aspects and their youngest child. Did you answer such questions for us last year ?<br>पिछले साल भी हमने आपके समूह की कुछ औरतों से गर्भावस्था और दुसरे स्वास्थ्य के बारें में और सबसे छोटे बच्चे के बारें में सवाल पूछें थे\ क्या आपने ऐसे सवालों के जवाब दिए थे पिछले साल?	Yes हां<br>No नहीं	1<br>2

Thank you for your time. आपके समय के लिए धन्यवाद
